# Characterizing belief bias in syllogistic reasoning: A hierarchical Bayesian meta-analysis of ROC data

**DOI:** 10.3758/s13423-018-1460-7

**Published:** 2018-06-25

**Authors:** Dries Trippas, David Kellen, Henrik Singmann, Gordon Pennycook, Derek J. Koehler, Jonathan A. Fugelsang, Chad Dubé

**Affiliations:** 1 Center for Adaptive Rationality, Max Planck Institute for Human Development, Berlin, Germany; 2 Syracuse University, Syracuse, NY USA; 3 University of Zurich, Zurich, Switzerland; 4 Yale University, New Haven, CT USA; 5 University of Waterloo, Waterloo, ON Canada; 6 University of South Florida, Tampa, FL USA

**Keywords:** Deductive reasoning, Syllogisms, Belief bias, Signal detection theory, Hierarchical Bayesian, Meta-analysis

## Abstract

The belief-bias effect is one of the most-studied biases in reasoning. A recent study of the phenomenon using the *signal detection theory* (SDT) model called into question all theoretical accounts of belief bias by demonstrating that belief-based differences in the ability to discriminate between valid and invalid syllogisms may be an artifact stemming from the use of inappropriate linear measurement models such as analysis of variance (Dube et al., *Psychological Review*, 117(3), 831–863, 2010). The discrepancy between Dube et al.’s, *Psychological Review*, 117(3), 831–863 (2010) results and the previous three decades of work, together with former’s methodological criticisms suggests the need to revisit earlier results, this time collecting confidence-rating responses. Using a hierarchical Bayesian meta-analysis, we reanalyzed a corpus of 22 confidence-rating studies (*N* = 993). The results indicated that extensive replications using confidence-rating data are unnecessary as the observed receiver operating characteristic functions are not systematically asymmetric. These results were subsequently corroborated by a novel experimental design based on SDT’s *generalized area theorem*. Although the meta-analysis confirms that believability does not influence discriminability unconditionally, it also confirmed previous results that factors such as individual differences mediate the effect. The main point is that data from previous and future studies can be safely analyzed using appropriate hierarchical methods that do not require confidence ratings. More generally, our results set a new standard for analyzing data and evaluating theories in reasoning. Important methodological and theoretical considerations for future work on belief bias and related domains are discussed.

The ability to draw necessary conclusions from given information constitutes one of the building blocks of knowledge acquisition. Without deduction, there would be no science, no technology, and no modern society (Johnson-Laird & Byrne, 1991). Over acentury of research has demonstrated that people can reason deductively, albeit imperfectly so (e.g., Störring, 1908; Wilkins, 1929). One key demonstration of the imperfect nature of deduction is aphenomenon known as *belief bias*, which has inspired an impressive amount of research and has been considered to be akey explanandum for any viable psychological theory of reasoning (for reviews, see Dube et al., 2010; Evans, 2002; Klauer et al., 2000). Consider the following syllogism (Markovits & Nantel, 1989):

*All flowers have petals.*

*All roses have petals.*

*Therefore, all roses are flowers.*

This syllogism is logically invalid, as the conclusion (i.e., the sentence beginning with “Therefore”) does not necessarily follow from the two premises, assuming the premises are true (i.e., the conclusion is possible, but not necessary). However, the fact that this syllogism’s conclusion states something consistent with real-world knowledge leads many individuals to endorse it as logically valid. More generally, syllogisms with believable conclusions are more often endorsed than structurally identical syllogisms that include unbelievable conclusions instead (e.g., “*no roses are flowers*”). At the heart of the belief bias effect is the interplay between individuals’ attempts to rely on the rules of logic and their general tendency to incorporate prior beliefs into their judgments and inferences (e.g., Bransford & Johnson, 1972; Cherubini et al., 1998; Schyns & Oliva, 1999). Although a reliance on prior belief is believed to be desirable and adaptive in many circumstances (Skyrms, 2000), it can be detrimental in cases where the goal is to assess the form of the arguments (e.g., in a court of law). Moreover, beliefs are often misguided and logical reasoning is necessary to determine if and when this is the case.

These detriments are likely to be far reaching in our lives, as highlighted by early work focusing on the social-psychological implications of belief bias (e.g., Feather, 1964; Kaufmann & Goldstein, 1967). Batson (1975), for example, found that presenting evidence that contradicts stated religious belief sometimes increases the intensity of belief. Motivated reasoning effects of this sort have been reported in hundreds of studies (Kunda, 1990), including, appropriately, on the Wason selection task (Dawson et al., 2002). Indeed, one of the foundational observations in the reasoning literature is the tendency for people to confirm hypotheses rather than disconfirm them (Wason, 1960, 1968; Wason and Evans, 1974), often referred to as confirmation bias (Nickerson, 1998) or attitude polarization (Lord et al., 1979). What makes belief bias notable is that, unlike in studies of motivated reasoning or attitude polarization, the beliefs that bias syllogistic reasoning are not of particular import to the reasoner (such as the “all roses are flowers” example above). Moreover, syllogistic reasoning offers a very clear logical standard by which to contrast the effect of belief bias. Thus, in a certain sense, developing a good account of belief bias in reasoning is foundational to understanding motivated reasoning and attitude polarization.

## Theoretical accounts of belief bias

In the last three decades, several theories have been proposed to describe how exactly beliefs interact with reasoning processes (e.g., Dube et al., 2010; Evans et al., 1983, 2001; Klauer et al., 2000; Markovits & Nantel, 1989; Newstead et al., 1992; Oakhill & Johnson-Laird, 1985; Quayle & Ball, 2000). For example, according to the *selective scrutiny* account (Evans et al., 1983), individuals uncritically accept arguments with a believable conclusion, but reason more thoroughly when conclusions are unbelievable. In contrast, proponents of a *misinterpreted necessity* account (Evans et al., 1983; Markovits & Nantel, 1989; Newstead et al., 1992) argue that believability only plays a role after individuals have reached conclusions that are consistent with, but not necessitated by, the premises (as in the example above).

Alternatively, *mental-model theory* (Johnson-Laird, 1983; Oakhill & Johnson-Laird, 1985) proposes that individuals evaluate syllogisms by generating mental representations that incorporate the premises. When the conclusion is consistent with one of these representations, the syllogism tends to be perceived as valid. However, when the conclusion is seen as unbelievable, the individual is assumed to engage in the creation of alternative mental representations that attempt to refute the conclusion (i.e., counterexamples). Only when a model is found wherein the (unbelievable) conclusion is consistent with these alternative representations, is the syllogism perceived to be valid.

Another account, *transitive-chain theory* (Guyote & Sternberg, 1981) proposes that reasoners encode set-subset relations between the terms of the syllogism inspired by the order in which said terms are encountered when reading the syllogism. These mental representations are then combined according to a set of matching rules with different degrees of exhaustiveness. The theory predicts that unbelievable contents add an additional burden to this information processing, leading to worse performance compared to syllogisms with believable contents.

Yet another account, *selective processing theory* (Evans et al., 2001), proposes that individuals use a conclusion-to-premises reasoning strategy. Participants are assumed to first evaluate the believability of the conclusion, after which they conduct a search for additional evidence. Believable conclusions trigger a search for confirmatory evidence, whereas unbelievable conclusions induce a disconfirmatory search. For valid problems the conclusion is consistent with all possible representations of the premises, so believability will not have a large effect on reasoning. By contrast, for indeterminately invalid problems a representation which is inconsistent with the premises can typically be found with a disconfirmatory search, leading to increased logical reasoning accuracy for unbelievable problems. Most recently, the model has been extended to predict that individual differences in thinking ability mediate these effects, such that more able thinkers are more likely to be influenced by their prior beliefs (Stupple et al., 2011; Trippas et al., 2013).

This brief description does not exhaust the many theoretical accounts proposed in the literature, each of them postulating distinct relationships between reasoning processes and prior beliefs (e.g., Newstead et al., 1992; Quayle & Ball, 2000; Polk & Newell, 1995; Thompson et al., 2003; for reviews see Dube et al., 2010; Klauer et al., 2000). However, irrespective of the precise interplay between beliefs and reasoning processes, a constant feature of these theories is that the ability to discriminate between logically valid and invalid syllogisms is predicted to be higher when conclusions are unbelievable (although the opposite prediction has also been made by transitive-chain theory). In sum, virtually all theories propose that beliefs have *some* effect on reasoning ability, the latter having been operationalized in terms of the ability to discriminate between valid and invalid syllogisms. In this manuscript we test if believability affects discriminability using a mathematical model based on signal detection theory. Before describing this model in detail, it is important to consider the motivation behind this quite prevalent assumption.

The experimental design most commonly used in modern studies on the belief bias was popularized by the seminal work of Evans et al., (1983). They used a $2\times 2$ design that orthogonally manipulated the logical status of syllogisms (*Logic*: valid *vs.* invalid syllogisms) along with the believability of the conclusion (*Belief*: believable vs. unbelievable syllogisms) while controlling for a number of potential confounds concerning the structure of syllogisms (e.g., figure and mood; for a review, see Khemlani & Johnson-Laird, 2012). Based on this Logic $\times $ Belief experimental design, one can compare the endorsement rates (using binary response options “valid” and “invalid”) associated with the different levels of each factor. Table 1 provides a summary of this design.

**Table 1:** The design of Evans et al. (1983, Experiment 1), example syllogisms, and endorsement rates

	Conclusion	
Syllogism	Believable	Unbelievable
Valid	No cigarettes are inexpensive.	No addictive things are inexpensive.
	Some addictive things are inexpensive.	Some cigarettes are inexpensive.
	Therefore, some addictive things are not	Therefore, some cigarettes are not addictive.
	cigarettes.	
	P(“valid”) = .92	P(“valid”) = .46
Invalid	No addictive things are inexpensive.	No cigarettes are inexpensive.
	Some cigarettes are inexpensive.	Some addictive things are inexpensive.
	Therefore, some addictive things are not	Therefore, some cigarettes are not addictive.
	cigarettes.	
	P(“valid”) = .92	P(“valid”) = .08

The endorsement rates obtained with such a $2\times 2$ design can be decomposed in terms of the contributions of logical validity (i.e., logic effect), conclusion believability (i.e., belief effect), and their interaction, as would be done with a *linear model* such as multiple regression. Taking Table 1 as an example, there is an effect of logical validity, with valid syllogisms being more strongly endorsed overall than their invalid counterparts ((.92 + .46)/2 − (.92 + .08)/2 > 0). There is also an effect of conclusion believability, as syllogisms with believable conclusions were endorsed at a much greater rate than syllogisms with unbelievable conclusions ((.92 + .92)/2 − (.46 + .08)/2 > 0). Finally, there is an interaction between validity and believability (Logic $\times $ Belief interaction): the difference in endorsement rates between valid and invalid syllogisms is much smaller when conclusions are believable than when they are unbelievable ((.92 − .92) − (.46 − .08) = −.38). At face value, the negative interaction emerging from these differences suggests that individuals’ reasoning abilities are reduced when dealing with syllogisms involving believable conclusions (although the effect is typically interpreted the other way around, such that people reason better when syllogisms have unbelievable conclusions; e.g., Lord et al., 1979). Since Evans et al., (1983), the interaction found in Logic $\times $ Belief experimental designs like the one illustrated in Table 1 is usually referred to as the *interaction index*.

Overall, these results suggest three things: First, that individuals can discriminate valid from invalid arguments, albeit imperfectly (i.e., individuals can engage in deductive reasoning). Second, that people are more likely to endorse syllogisms as valid if their conclusions are believable (i.e., consistent with real-world knowledge) than if they are not. Third, that people are more likely to discriminate between logically valid and invalid conclusions when those conclusions are unbelievable. In contrast with the main effects of logical validity and believability, which are not particularly surprising from a theoretical point of view (Evans and Stanovich, 2013), the Logic $\times $ Belief interaction has been the focus of many research endeavors and is considered to be a basic datum that theories of the belief bias need to explain in order to be viable (Ball et al., 2006; Evans & Curtis-Holmes, 2005; Morley et al., 2004; Newstead et al., 1992; Quayle & Ball, 2000; Shynkaruk & Thompson, 2006; Stupple & Ball, 2008; Thompson et al., 2003; Roberts & Sykes, 2003).

Researchers’ reliance on the interaction index to gauge changes in reasoning abilities was the target of extensive criticisms by Klauer et al., (2000) and Dube et al., (2010). Both Klauer et al. and Dube et al. demonstrated that the linear-model-based approach used to derive the interaction index hinges on questionable assumptions regarding the way endorsement rates for valid and invalid syllogisms relate to each other. They argued that any analysis of the belief-bias effect rests upon some theoretical measurement model whose core assumptions need to be checked before any interpretation of the results can be safely made. Using extended experimental designs that go beyond the traditional Logic $\times $ Belief design (e.g., introducing response-bias manipulations, payoff matrices, the use of confidence-rating scales) and including extensive model-validation tests, Klauer et al. and Dube et al. showed that the assumptions underlying the linear-model-based approach are incorrect, raising doubts about studies that take the interaction index as a direct measure of change in reasoning abilities. But whereas Klauer et al.’s results were still in line with the notion that conclusion believability affects the ability to discriminate between valid and invalid syllogisms, the work by Dube et al., (2010) argued that conclusion believability does not affect individuals’ discrimination abilities at all. Instead, their account suggests that conclusion believability affects only the general tendency towards endorsing syllogisms as valid (irrespective of their logical status). Dube et al.’s results are therefore at odds with most theories of deductive reasoning (but see Klauer & Kellen, 2011 and the response by Dube et al., 2012).^1^

The results of Dube et al., (2010) can be interpreted as calling for the establishment of a new standard for methodological and statistical practices in the domain of syllogistic reasoning and deductive reasoning more generally (Heit and Rotello, 2014). Simply put, the use of flawed reasoning indices should be abandoned in favor of extended experimental designs that allow for the testing of the assumptions underlying the data analysis method. Specifically, their simulation and experimental results suggest moving from requesting binary judgments of validity to the use of experimental designs that request participants to report their judgments using a confidence-rating scale (e.g., a six-point scale from *1: very sure invalid* to *6: very sure valid*). These data can then be used to obtain *receiver operating characteristic* (ROC) functions and fit *signal detection theory* (SDT), a prominent measurement model in the literature that has been successfully applied in many domains (e.g., memory, perception; for introductions, see Green & Swets, 1966; Kellen & Klauer, 2018; Macmillan & Creelman, 2005). The parameter estimates provided by the SDT model can inform us on the exact nature of the observed differences in endorsement rates. Although experimental data from previous studies could potentially be reanalyzed with a version of SDT—known as the equal variance SDT model—which does not require confidence ratings, there is evidence from simulations suggesting that reliance on this simpler version of SDT would hardly represent an improvement over the interaction index (Heit & Rotello, 2014): a more extensive version of SDT—known as the *unequal variance* SDT model–appears to be necessary.^2^

Taken at face value, the implications of Dube et al., (2010) work are severe and far-reaching, as they suggest that the majority of the work published in the last 30 years on belief bias must be *conducted anew* with extended experimental designs in order to determine whether the original findings can be validated with SDT (see also Rotello et al., 2015, for a similar suggestion in other psychological domains). However, there are legitimate concerns that Dube et al.’s results could have been distorted by their reliance on aggregated data. And if this is indeed the case, then it is possible that the implications are less severe. Aggregation overlooks the heterogeneity that is found among participants and stimuli. The problems associated with data aggregation, which have been long documented in the psychological literature (e.g., Estes, 1956; Estes & Todd Maddox, 2005; Judd et al., 2012), also hold for the case of ROC data (e.g., DeCarlo, 2011; Malmberg & Xu, 2006; Morey et al., 2008; Pratte & Rouder, 2011; Pratte et al., 2010). But to the best of our knowledge, these concerns have only been mentioned before in the context of the belief bias effect (e.g., Dube et al., 2010; Klauer et al., 2000), but have not been directly addressed. In order to address these concerns head on, we relied on a hierarchical Bayesian implementation of the unequal variance SDT model that takes into account differences across stimuli, participants, and studies. Using this model, we were able to conduct a meta-analysis over a confidence-rating ROC data corpus comprised of over 900 participants coming from 22 studies. To the best of our knowledge, this corpus contains the vast majority of published and unpublished research on belief bias in syllogistic reasoning *for which confidence ratings were collected*. The results obtained from this meta-analysis will allow us to answer the following questions:

- Can the equal variance SDT model provide a sensible account of the data, dimissing the need for extended experimental designs?
- Does the believability of conclusions affect people’s ability to discriminate between valid and invalid syllogisms?

In addition to these main questions, we will also briefly revisit the evidence for the role of individual differences in belief bias for a subset of the data for which this information is available. Our results discussed below show that the confidence-rating data are very much in line with the predictions made by the equal variance SDT model which can be applied without the availability of confidence ratings, suggesting that previously published belief bias studies can be reanalyzed using a probit or logit regression. The results also suggest that despite the heterogeneity found among participants and stimuli, the believability of conclusions does not generally affect people’s ability to discriminate between valid and invalid syllogisms when considered across the entire corpus, partially confirming (Dube et al., 2010) original account. However, a closer inspection using individual covariates suggest a relationship between people’s reasoning abilities and the way they are affected by beliefs, as suggested by Trippas et al. (2013, 2014, 2015). Altogether, these results suggest that syllogistic reasoning should be analyzed using hierarchical statistical methods together with additional individual covariates. In contrast, the routine collection of confidence ratings with the aim of modeling data, while certainly a possibility, is by no means necessary.

The remainder of this manuscript is organized as follows: First, we will review some of the problems associated with traditional analyses of the belief-bias effect based on a linear model, followed by an introduction to SDT and the analysis of ROC data. We then turn to the risks associated with the aggregation of heterogeneous data across participants and stimuli and how they can be sidestepped through the use of hierarchical Bayesian methods. In addition to the meta-analysis, we report a series of validation checks that corroborate our findings. Next, we present data from a new experiment using a K-alternative forced choice task which corroborates the main conclusion from our meta-analysis. Finally, we discuss potential future applications for the data-analytic methods used here and theoretical implications for belief bias.

## Implicit linear-model assumptions and SDT-based criticisms

In order to understand the problems associated with the linear-model approach, it is necessary to describe in greater detail how it provides a *linear decomposition* of the observed endorsement rates in terms of simple effects and interactions. The probability of an endorsement (responding “valid”) in a typical $2\times 2$ experimental design with factors of Logic (*L*: invalid = -1; valid = 1) and Belief (*B*: unbelievable = -1, believable = 1) is given by:

1 $$ P(\text{``valid''} | L, B) = \beta_{0} + L\beta_{L} + B\beta_{B} + LB\beta_{LB}, $$

where parameters $\beta _{0}$, $\beta _{L}$, $\beta _{B}$, and $\beta _{LB}$ denote, in order, the intercept (i.e., the grand mean propensity to endorse syllogisms), the main effects of Logic and Belief (*β*_*L*_ and $\beta _{B}$ actually only represent $\frac {1}{2}$ times the main effects), and the interaction between the latter two (*LB* = *L* × *B*). It is assumed that there is a linear relationship between a latent construct, which we will refer to as “reasoning ability”, and the effects of Logic and Belief (Evans et al., 1983; Evans and Curtis-Holmes, 2005; Newstead et al., 1992; Roberts & Sykes, 2003; Stupple & Ball, 2008).

**Fig. 1:**
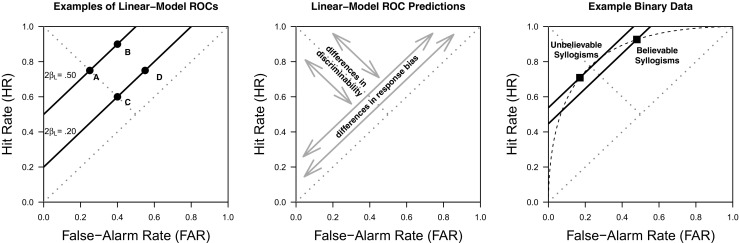
*Left Panel*: Examples of ROCs predicted by the linear model. *Center Panel*: Illustration of how differences in response bias and discriminability in the linear model are expressed in terms of ROCs. *Right Panel*: Example of data that according to the linear model imply differences in discriminability for believable and unbelievable syllogisms, but could be described in terms of different response biases if the predicted ROC was curvilinear

A first problem with this linear-model approach is the fact that it does not respect the nature of the data it attempts to characterize. The parameters can take on any values, enabling predictions that are outside of the unit interval in which proportions are represented. Another concern relates with the way that the indices/parameters are typically interpreted, in particular the interaction index $\beta _{LB}$. Specifically, negative interactions like the one described in Table 1 do not necessarily imply a diminished reasoning ability but may simply reflect the existence of a *non-linear relationship* between this latent construct and the factors of the experimental design (see Wagenmakers et al., 2012). This point was made by Dube et al., (2010), who highlighted the fact that the relationship between the latent reasoning ability and the factors of the experimental design can be assessed by means of receiver operating characteristic (ROC) functions. In the case of syllogistic reasoning, ROCs plot the endorsement rates of invalid syllogisms (false alarm rate; FAR) on the *x*-axis, and the endorsement rates of valid syllogisms (hit rate; HR) on the *y*-axis (e.g., Fig. 1).

First, consider an experimental design without believability manipulation in which only the syllogisms’ logical validity is manipulated. According to the linear model described in Eq. 1, predicted hit and false-alarm rates are given by:

2 $$\begin{array}{@{}rcl@{}} \text{FAR} = P(\text{``valid''} | L = -1) &=& \beta_{0} - \beta_{L}, \end{array} $$

3 $$\begin{array}{@{}rcl@{}} \text{HR} = P(\text{``valid''} | L = 1) &=& \beta_{0} + \beta_{L}. \end{array} $$

It is easy to see that the hit rate and false-alarm rate are related in a linear fashion. Consider for example an observation with $\beta _{0} = .5$ and $\beta _{L} = .25$. This results in a FAR = .25 and HR = .75 (i.e., a logic effect of .5), denoted *A* in Fig. 1 (left panel). Now consider we increase the mean endorsement rate, but not the effect of validity, by .15 such that $\beta _{0} = .65$ and $\beta _{L} = .25$. This results in a FAR = .4 and HR = .9, denoted *B*. Next, consider we manipulate the effect of validity, but not the mean endorsement rate, in reference to point *A* such that $\beta _{0} = .5$ and $\beta _{L} = .1$. This gives us FAR = .4 and HR = .6 denoted as *C*. Finally, we manipulate both the mean endorsement and the effect of validity in reference to point *A* such that $\beta _{0} = .65$ and $\beta _{L} = .1$. This gives us FAR = .55 and HR = .75 denoted *D*. As can be easily seen, *A* and *B* are connected by a linear ROC with unit slope (i.e., slope = 1) and ROC-intercept equal to the main effect of logic, $2\beta _{L}$ = .5. Likewise, *C* and *D* are connected by a linear ROC with unit slope and ROC-intercept $2\beta _{L}$ = .2. This allows two simple conclusions (see center panel of Fig. 1): (1) Manipulations that affect the *discriminability* between valid and invalid syllogism affect the ROC-intercept and create different ROC lines. (2) All data points resulting from manipulations that affect the average endorsement rate, but not the ability to discriminate between valid and invalid syllogism, lie on the same linear ROC with unit slope. Manipulations within one item class (e.g., believable syllogisms) that leave the discriminability unaffected are also called *response bias*.

Now consider a full experimental design with in which both validity and believability are manipulated. This gives us the following linear model:

4 $$\begin{array}{@{}rcl@{}} \text{FAR}_{\text{unbelievable}} = P(\text{``valid''} | L = -1, B = -1) &=& \beta_{0} - \beta_{L} - \beta_{B} + \beta_{LB}, \end{array} $$

5 $$\begin{array}{@{}rcl@{}} \text{HR}_{\text{unbelievable}} = P(\text{``valid''} | L = 1, B = -1) &=& \beta_{0} + \beta_{L} - \beta_{B} - \beta_{LB}, \end{array} $$

6 $$\begin{array}{@{}rcl@{}} \text{FAR}_{\text{believable}} = P(\text{``valid''} | L = -1, B = 1) &=& \beta_{0} - \beta_{L} + \beta_{B} - \beta_{LB}, \end{array} $$

7 $$\begin{array}{@{}rcl@{}} \text{HR}_{\text{believable}} = P(\text{``valid''} | L = 1, B = 1) &=& \beta_{0} + \beta_{L} + \beta_{B} + \beta_{LB}. \end{array} $$

From these equations it is easy to see that in the absence of an interaction (i.e., $\beta _{LB} = 0$) all data points would fall on the same unit-slope ROC. The only change for each pair of FAR and HR for each believability condition is that the same value is either subtracted (for unbelievable syllogisms) or added (for believable syllogisms). And adding a constant to both *x*- and *y*-coordinates only moves a point along a unit slope. In contrast, what the interaction does is to alter the Logic effect for each believability condition; it creates separate ROCs. For example, negative values of $\beta _{LB}$ increase the Logic effect for unbelievable syllogisms and decrease the Logic effect for believable syllogisms. Hence, if $\beta _{LB} \neq 0$ the two believability conditions would fall on two separate ROCs, with the ROC for unbelievable syllogisms above the one for believable syllogisms for negative values of $\beta _{LB}$.

The assumption that ROCs are linear (with slope 1) is questionable, given that the ROCs obtained across a wide range of domains tend to show a *curvilinear* shape (Green and Swets, 1966; Dube & Rotello, 2012); but see Kellen et al., (2013). The possibility of ROCs being curvilinear is problematic for the linear model given that it can misinterpret differences in response bias as differences in discriminability. For example, in the right panel of Fig. 1 we illustrate a case in which the discriminability for believable syllogisms is found to be lower than for unbelievable syllogisms (negative interaction index $\beta _{I}$), despite the fact that according to SDT (dashed curve) the observed ROC points can be understood as differing in terms of response bias alone. Moreover, potentially curvilinear ROC shapes are theoretically relevant given that they are considered a signature prediction of signal detection theory (SDT).

### Signal detection theory

According to the SDT model, the validity of syllogisms is represented on a continuous latent-strength axis, which in the present context we will simply refer to as *argument strength* (Dube et al., 2010). The argument strength of a given syllogism can be seen as the output of a participant’s reasoning processes (e.g., Chater & Oaksford, 1999; Oaksford & Chater, 2007). A syllogism is endorsed as valid whenever its argument strength is larger than a *response criterion* $\tau $. When the syllogism’s argument strength is smaller than the response criterion, the syllogism is deemed as invalid. This response criterion is assumed to reflect an individual’s general bias towards endorsement: more lenient individuals will place the response criterion at lower argument-strength values than individuals who tend to be quite conservative in their endorsements. Different criteria have consequences for the amount of correct and incorrect judgments that are made: for example, conservative criteria lead to less false alarms than their liberal counterparts but also lead to less hits.

**Fig. 2:**
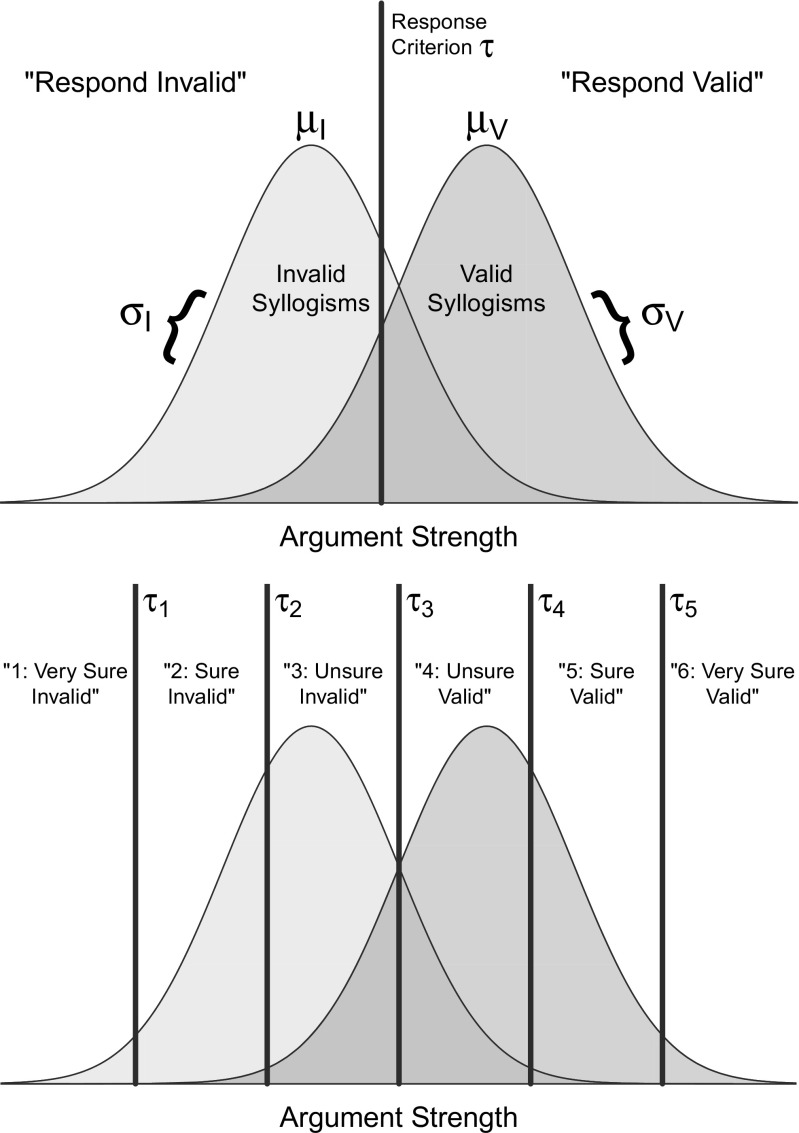
Illustration of the Gaussian SDT model. The *top panel* shows argument strength distributions for valid and invalid syllogisms (and their respective parameters) in the case of binary choices. The *bottom panel* illustrates the same model in the case of responses in a six-point confidence-rating scale (for clarity, some parameters and labels are omitted here)

A common assumption in SDT modeling is that the argument strengths of valid and invalid syllogisms can be described by Gaussian distributions with some mean $\mu $ and standard deviation $\sigma $. These distributions reflect the expectation and variability in argument strength that is associated with valid and invalid syllogisms. The farther apart these two distributions are—that is, the smaller their overlap—the better individuals are in discriminating between valid and invalid syllogisms. Figure 2 (top panel) illustrates a pair of evidence strength distributions associated with valid and invalid syllogisms and a response criterion.

From the postulates of SDT, it follows that the probabilities of endorsing valid (V) and invalid (I) syllogisms correspond to the areas of the two distributions that are to the right side of the response criterion $\tau $. Formally,

8 $$\begin{array}{@{}rcl@{}} \text{FAR} = P(\text{``valid''} | L = -1) &=& {\Phi}\left( \frac{-\tau + \mu_{I}}{\sigma_{I}} \right), \end{array} $$

9 $$\begin{array}{@{}rcl@{}} \text{HR} = P(\text{``valid''} | L = 1) &=& {\Phi}\left( \frac{-\tau + \mu_{V}}{\sigma_{V}}\right), \end{array} $$

where $\mu _{I}$ and $\sigma _{I}$ correspond to the mean and standard deviation of the distribution for invalid syllogisms, and $\mu _{V}$ and $\sigma _{V}$ are their counterparts for valid syllogisms. The function ${\Phi }(\cdot )$ corresponds to the cumulative distribution function of the standard Gaussian distribution, which translates values from a latent argument-strength scale (with support across the real line) onto a probability scale between 0 and 1. This translation ensures that the model predictions are in line with the nature of the data they attempt to characterize.

**Fig. 3:**
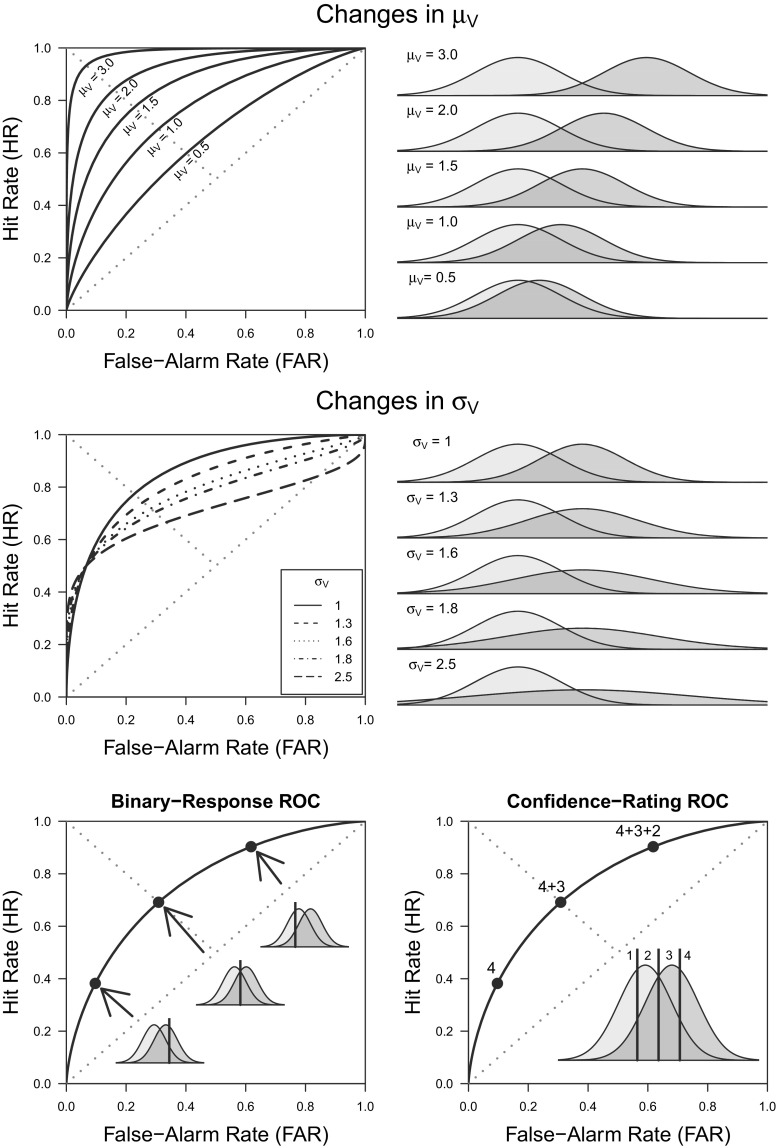
Illustration of SDT predictions. The *top row* illustrates how ROC predictions change across values of *μ*_*V*_ (with *μ*_*I*_ = 0 and *σ*_*I*_ = 1). The *middle row* illustrates how ROC predictions change across values of *σ*_*V*_. The *bottom-left panel* shows how hits and false alarms change due changes in the response criterion. The *bottom-right panel* illustrates how confidence-rating ROCs (4-point scale) are based on the cumulative probabilities associated with the different confidence responses

The lower-left panel of Fig. 3 show how the differences in the position of the response criteria are expressed in terms of hits and false alarms. Note that the illustration of the latent distributions postulated by SDT in Fig. 2 does not specify the origin nor the unit of the latent argument–strength axis. In order to establish both the origin and unit, it is necessary to fix some of the model’s parameters. It is customary to fix the standard deviation $\sigma _{I}$ to 1 and the mean $\mu _{I}$ to either 0 or $-\mu _{V}$. When these restrictions are imposed, one can simply focus on the parameters for valid syllogisms, $\mu _{V}$ and $\sigma _{V}$ (but alternative restrictions are possible, one of which will be used later on). It is important to note that these scaling restrictions do not affect the performance of the model in any way. The overall ability to discriminate between valid and invalid syllogisms can be summarized by an adjusted distance measure $d_{a}$ (Simpson and Fitter, 1973):

10 $$ d_{a} = \sqrt{2}\times\frac{\mu_{V} - \mu_{I}}{\sqrt{{\sigma_{V}^{2}} + {\sigma_{I}^{2}}}} $$

If one would assume the parametrization in which $\mu _{I} = -\mu _{V}$ and $\sigma _{I} = 1$, the similarity between Eqs. 2 and 3 of the linear model and Eqs. 8 and 9 of the SDT model becomes obvious. Response criterion $\tau $ plays the same role as the intercept $\beta _{0}$, in that both determine the endorsement rate for invalid syllogisms. Meanwhile, the mean $\mu _{V}$ plays the role of $\beta _{L}$ by capturing the effect of Logic (*L*)— i.e., a reflection of reasoning aptitude, with a value of 0 suggesting an inability to discriminate between valid and invalid arguments. From this standpoint, the differences between the linear model and SDT models essentially boil down to the latter assuming a parameter $\sigma _{V}$ that modulates how the response-criterion $\tau $ affects the hit rate, and the use of the non-linear function ${\Phi }(\cdot )$ which translates the latent argument-strength values into manifest response probabilities (DeCarlo, 1998) and maps the real line onto the probability scale. Although these differences may seem minor or even pedantic, they are highly consequential, as they ultimately lead both models to yield rather distinct interpretations of the same data (see the right panel of Fig. 1). Figure 3 shows ROCs generated by the SDT model under different parameter values: as the ability to discriminate between valid and invalid syllogisms increases (e.g., $\mu _{V}$ increases), so does the area under the ROC. Moreover, parameter $\sigma _{V}$ affects the symmetry/asymmetry of the ROC relative to the negative diagonal, with ROCs only being symmetrical when $\sigma _{V} = \sigma _{I}$. Note that all these ROCs are *curvilinear*, in contrast with the unit-slope *linear* ROCs predicted by the ANOVA model (compare with the left panel of Fig. 1).

Dube et al., (2010) showed that the linear model can produce an inaccurate account of the data simply due to the mismatch between the model’s predictions and the observed ROC data. Specifically, if the ROCs are indeed curved as predicted by SDT, then the linear model is likely to incorrectly interpret these data as evidence for a difference in discrimination. This difference in discrimination would be captured by a statistically significant interaction index. For example, consider the right panel of Fig. 1, which illustrates a case where the hit and false-alarm rates observed across believability conditions all fall on the same curved ROC, a pattern indicating that these conditions only differ in terms of the response bias being imposed (i.e., these rates reflect the same ability to discriminate between valid and invalid syllogisms): the linear model cannot capture both ROC points in the same unit-slope line, which yields the erroneous conclusion that there is a difference in the level of valid/invalid discrimination for believable and unbelievable syllogisms (a difference captured by the interaction index $\beta _{I}$). Note that this erroneous conclusion does not vanish by simply collecting more data—in fact, additional data will only reinforce the conclusion, an aspect that can lead researchers to a false sense of reassurance. Rotello et al., (2015) discussed how researchers tend to be less critical of the interpretation of their measurements when they are replicated on a regular basis. Given that negative interaction indices are regularly found in syllogistic-reasoning studies, very few researchers have considered evaluating the measurement model that underlies this index (the exceptions are Dube et al., 2010; Klauer et al., 2000).

In order to assess the shape of syllogistic-reasoning ROCs and compare them with the predictions coming from the linear and SDT models, Dube et al., (2010) relied on an extended experimental design in which confidence-rating judgments were also collected. In the SDT framework, confidence ratings can be modeled via a set of ordered response criteria (for details, see Green & Swets, 1966; Kellen & Klauer, 2018). For instance, according to SDT, in the case of a six-point scale ranging from “1: *very sure invalid*” to “6: *very sure valid*”, the probability of a confidence rating $1 \leq k \leq 6$ can be obtained by establishing five response criteria $\tau _{k}$, with $\tau _{k-1} \leq \tau _{k}$ for all $ 2 \leq k \leq 6$, as illustrated in the lower panel of Fig. 2:

11 $$\begin{array}{@{}rcl@{}} P(\text{``}k\text{''} | \text{Logic} = -1) = \left\{\begin{array}{llll} {\Phi}\left( \frac{\tau_{k} - \mu_{I}}{\sigma_{I}} \right), \;\; \text{ when} \;\;k = 1,\\ {\Phi}\left( \frac{\tau_{k} - \mu_{I}}{\sigma_{I}} \right) - {\Phi}\left( \frac{\tau_{k-1} + \mu_{I}}{\sigma_{I}} \right), \;\; \text{ when} \;\;2 \leq k \leq 5, \\ 1-{\Phi}\left( \frac{\tau_{k-1} - \mu_{I}}{\sigma_{I}} \right), \;\;\text{ when} \;\;k = 6, \end{array}\right. \end{array} $$

12 $$\begin{array}{@{}rcl@{}} P(\text{``}k\text{''} | \text{Logic} = 1) = \left\{\begin{array}{ll} {\Phi}\left( \frac{\tau_{k} - \mu_{V}}{\sigma_{V}} \right), \;\; \text{ when} \;\;k = 1,\\ {\Phi}\left( \frac{\tau_{k} - \mu_{V}}{\sigma_{V}} \right) - {\Phi}\left( \frac{\tau_{k-1} - \mu_{V}}{\sigma_{V}} \right), \;\; \text{ when} \;\;2 \leq k \leq 5, \\ 1-{\Phi}\left( \frac{\tau_{k-1} - \mu_{V}}{\sigma_{V}} \right), \;\;\text{ when} \;\;k = 6, \end{array}\right. \end{array} $$

**Fig. 4:**
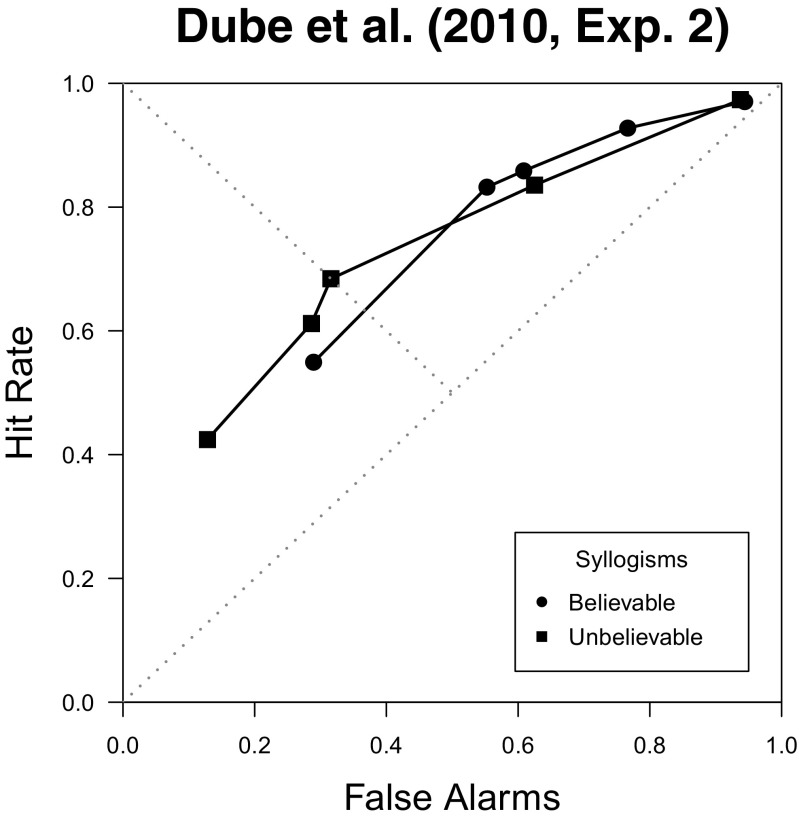
ROC data from Dube et al. (2010, Exp. 2)

Cumulative confidence probabilities can then be used to construct confidence-rating ROCs (for an example, see the bottom-right panel of Fig. 3). Dube et al.’s (2010) confidence-rating ROCs were found to be curvilinear, closely following the SDT model’s predictions. Moreover, Dube et al. showed that the belief-bias effect did not affect discriminability, in contrast with the large body of work based on the interaction index that attributed such an effect to differences in discriminability. Figure 4 provides a graphical depiction of this result, with both ROCs for believable and unbelievable syllogisms following a single monotonic curve. Overall, it turned out that the degree of overlap between the distributions for valid and invalid syllogisms was not affected by the believability of the conclusions. Moreover, Dube et al. showed that the linear-model-based approach tends to misattribute the belief-bias effect to individuals’ ability to discriminate between syllogisms, simply due to its failure to accurately describe the shape of the ROC. These issues were further fleshed out by Heit and Rotello (2014) in a series of simulations and experimental studies showing that different measures that do not hinge on ROC data tend to systematically mischaracterize the differences found between believable and unbelievable syllogisms.

## SDT’s point measure *d’*: A more efficient, equally valid approach?

Due to a general reliance on the interaction index, there is a real possibility that much of the literature on the belief bias effect is founded on an improper interpretation of an empirical finding. Ideally, this situation could be easily resolved by simply reanalyzing the existing binary data obtained from the commonly used Logic $\times $ Belief paradigm with an alternative SDT model that could provide parameter estimates with such data, to see if the conclusions regarding reasoning ability hold up. The equal-variance SDT (EVSDT) model, which fixes $\sigma _{V}$ and $\sigma _{I}$ to be equal to the same value seems like an ideal candidate in this respect as it is able to estimate discrimination (*μ*_*V*_) directly from a single pair of hit and false-alarm rates. This discrimination estimate is widely known in the literature as $d^{\prime }$ (Green & Swets, 1966). When, without loss of generality, we fix $\mu _{I} = 0$ and $\sigma _{V} =\sigma _{I} = 1$:

13 $$ d^{\prime} = {\Phi}^{-1}(\text{HR}) -{\Phi}^{-1}(\text{FAR})= -\tau + \mu_{V} + \tau = \mu_{V}, $$

where ${\Phi }^{-1}(\cdot )$ is the inverse of the Gaussian cumulative distribution function.

One important aspect of the EVSDT model is that it is formally equivalent to *probit regression*, with Logic and Belief factors (and their interaction):

14 $$ P(\text{``valid''} | L, B) = {\Phi} (\beta_{0} + L\beta_{L} + B\beta_{B} + LB\beta_{LB}). $$

A key difference between the linear model previously discussed (see Eq. 1) and probit regression is that the latter includes a *link function* ${\Phi } (\cdot )$ that maps the linear model onto a 0-1 probability scale (DeCarlo, 1998). If this simplified model is deemed appropriate, then one could keep relying on *a* logic $\times $ belief interaction index to assess the impact of beliefs on reasoning abilities.^3^

Like SDT, the simpler EVSDT model also predicts curvilinear ROCs, however they are all constrained to be *symmetrical* with respect to the negative diagonal. This additional constraint raises questions regarding the suitability of EVSDT: do the EVSDT’s predictions match the ROC data? And if not to which extent does this mismatch affect the characterization of the belief-bias effect? In other domains such as recognition memory and perception, ROCs have been found to be asymmetrical, with $\sigma _{V} > \sigma _{I}$ (see Dube & Rotello, 2012; Starns et al., 2012). When applied to these asymmetric ROCs, $d^{\prime }$ provides distorted results, with discriminability being overestimated in the presence of stricter response criteria, and underestimated for more lenient criteria (for an overview, see Verde et al., 2006). Similar results have been found in the case of syllogistic reasoning, with asymmetrical ROCs speaking strongly against the EVSDT model. Dube et al., (2010) found the restriction $\sigma _{V} = \sigma _{I}$ to yield predictions that systematically mismatch the ROC data.

These shortcomings were corroborated in a more comprehensive evaluation by Heit and Rotello (2014). They reported a simulation showing that, if anything, the use of $d^{\prime }$ only amounts to a small improvement over the interaction index. Specifically, data were generated via a bootstrap procedure and discrimination for syllogisms with believable and unbelievable conclusions were assessed with $d^{\prime }$ and the interaction index. Both measures were found to be strongly correlated and very often reached the same incorrect conclusion. The only difference was that $d^{\prime }$ led to incorrect conclusions slightly less often than the interaction index. Overall, the use of the EVSDT model and its measure $d^{\prime }$ does not seem to constitute a reasonable solution for the study of the belief-bias effect. These results suggest that researchers need to rely on extended designs (e.g., confidence ratings) whenever possible (Heit & Rotello, 2014, p. 90). But as it will be shown below, the dismissal of the EVSDT model and $d^{\prime }$ is far from definitive. In fact, it is entirely possible that this dismissal is the byproduct of an unjustified reliance on ROC data that aggregate responses across heterogeneous participants and stimuli.

## The problem of aggregating data from heterogeneous sources

One of the challenges experimental psychologists regularly face is the sparseness of data at the level of individuals as well as stimuli. Typically, one can only get a small number of responses from each participant, only have a small set of stimuli available, and can only obtain one response per participant-stimulus pairing. In the end, only very little data is left to work with. A typical solution to this sparseness problem consists of aggregating data across stimuli or participants. Although previous work has shown that although data aggregation is not without merits (Cohen et al., 2008), its use implies the assumption that that there are no differences between participants nor stimuli. In the presence of heterogeneous participants and stimuli, this assumption can lead to a host of undesirable effects. One classic demonstration of the risks of data aggregation in the social sciences is *Condorcet’s Paradox* (Condorcet, 1785), which demonstrates how preferences (e.g., between political candidates) aggregated across individuals might not reflect properties that hold for any individual. In this specific case, it is shown that aggregated preferences often violate a fundamental property of rational preferences known as transitivity (e.g., if option A is preferred to B, and option B is preferred to C, then option A is preferred to C), even though all of the aggregated individual preferences were actually transitive (for a discussion, see Regenwetter et al., 2011).

In the case of traditional data-analytic methods such as linear models, the aggregation of data coming from heterogeneous participants and stimuli often leads to distorted results and severely inflated type I errors. These distortions can also compromise the replication and generalization of findings (for an overview, see Judd et al., 2012). Other approaches which do not rely on aggregation, for instance analyzing the data for each participant individually prior to summarizing them, is also not ideal given that this approach may seriously inflate the probability of type 2 errors due to the data sparseness. The problems associated with data aggregation and pure individual-level analysis have led to a growing reliance on statistical methods that do not rely exclusively on either, but a compromise between both, effectively establishing a new standard in terms of data analysis (e.g., Baayen et al., 2008; Barr et al., 2013; Snijders & Bosker, 2012). Some of these methods have been adopted in recent work on probabilistic and causal reasoning (e.g., Haigh et al., 2013; Rottman & Hastie, 2016; Singmann et al., 2016, 2014; Skovgaard-Olsen et al., 2016), but these methods have not been applied to the study of the measurement assumptions underlying belief bias. For example, for a very long time it was established in the literature that the effects of practice in cognitive and motor skills were better characterized by a power function than by an exponential function (Newell et al., 1981). However, this finding was based on functions aggregated across participants. Later simulation work showed that when agregated across participants, exponential practice functions were better accounted for by a power function (Anderson & Tweney, 1997; Heathcote et al., 2000). In an analysis involving data from almost 500 participants, Heathcote et al. showed that non-aggregated data were better described by an exponential function, a result that demonstrates how a reliance on aggregate data can lead researchers astray for several decades. Another example can be found in the domain of cognitive neuroscience, where it is common practice to aggregate across multiple participants’ fMRI-data. In contrast to the prevailing assumption in the field, individual patterns of brain activity are not exclusively driven by external or measurement noise, but are potentially linked to systematic inter-individual differences in strategy use (Miller et al., 2002).

### Aggregating across heterogeneous stimuli

**Fig. 5:**
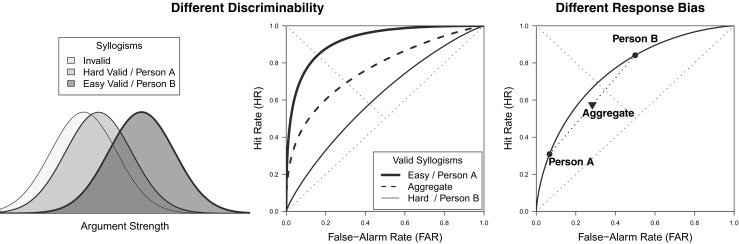
Illustration of the effects caused by the aggregation of responses across heterogeneous participants and stimuli

Let us now describe some of the distortions that could be caused by the unaccounted presence of heterogeneous participants and stimuli (for similar scenarios, see Morey et al., 2008; Pratte et al., 2010): First, consider the judgments from a single individual who was requested to evaluate a list of valid and invalid syllogisms. Let us assume that these judgments are perfectly in line with the SDT model. Furthermore, assume that among the valid syllogisms, half were easy, $\mu _{V,\text {easy}}= 3$, and the other half were hard, $\mu _{V,\text {hard}}= 1$ (with $\mu _{I}= 0$). Moreover, assume that all argument-strength distributions have the same standard deviation, with $\sigma _{V,\text {easy}} = \sigma _{V,\text {hard}} = \sigma _{I} = 1$. These distributions are illustrated on the left panel of Fig. 5.

When the researcher must aggregate across easy and hard syllogisms because they cannot be differentiated a priori, one would hope to obtain parameter estimates that are in line with the average of the distributions’ parameters, namely $\mu _{V}= 2$ and $\sigma _{V}= 1$. Note that this average would respect the fact that all distributions have the same variances, yielding symmetric ROCs. Unfortunately, the parameter estimates one obtains from aggregating across stimuli does not produce such a result. Instead, the parameter estimates obtained underestimate $\mu _{V}$ and inflate $\sigma _{V}$. The problem here is that the average of both distributions will have a greater standard deviation than the average of $\sigma _{V,\text {easy}}$ and $\sigma _{V,\text {hard}}$. In this particular example, data aggregation led to an asymmetric ROC (see the center panel of Fig. 5) with estimates $\mu _{V}= 1.88$ and $\sigma _{V} = 1.32$.^4^ Based on these estimates, a researcher would erroneously conclude that ROCs are asymmetric and that one is required to estimate $\sigma _{V}$ (perhaps using a confidence-rating task) in order to accurately characterize the data. To make matters worse these distortions are asymptotic in the sense that they would not vanish by simply having more data. On the contrary, they only reinforce the distorted results. These results show that a scenario in which the rejection of EVSDT is driven by the use of heterogeneous stimuli is far from unlikely, given that there is substantial variability in the propensity to accept different syllogistic structures all classified as similarly complex (Evans et al.,, 1999). The presence of such asymptotic distortions is particularly troubling given that it can lead researchers to dismiss a large body of work in favor of new studies involving extended experimental designs.

### Aggregating across heterogeneous participants

We now turn to two examples involving the aggregation of judgments coming from two heterogeneous participants A and B. The first example is formally equivalent to the one just described in the subsection above (i.e., the left and center panels of Fig. 5 serve to illustrate it as well). Assume that participant A shows worse discriminability than (*μ*_*V*,*A*_ = 1) than participant B (*μ*_*V*,*B*_ = 3), with everything else being equal (again, $\mu _{0} = 1$, and $\sigma _{V,\text {easy}} = \sigma _{V,\text {hard}} = \sigma _{I} = 1$). Note that both participants’ ROCs are symmetrical. As in the case of heterogeneous stimuli, the aggregation of the data from these two individuals would lead to an asymmetric ROC and an inflated estimate of $\sigma _{V}$ (again, 1.32). In this scenario, the fact that one participant performs better than the other one is enough to distort the overall shape of the ROC. Once again, this possibility is far from unexpected in light of the fact that individual differences in reasoning ability are commonly found (Stanovich, 1999; Trippas et al., 2015).

The second example concerns differences in response bias, which can also produce distortions: For example, let us imagine two participants that have the same ability to discriminate between valid and invalid syllogisms, $\mu _{V}= 1$, but differ in terms of their response biases. Specifically, let us assume that participant A relies on a conservative criterion $\tau = 1.5$ (i.e., is less likely to endorse syllogisms), whereas participant B relies on the more lenient criterion $\tau = 0$ (i.e., is more likely to endorse syllogisms). The hit and false-alarm rate pairs for these two participants are (.31, .07) and (.84,.50), respectively. The pair obtained when aggregating both pairs, (.57, .28), is associated with $\mu _{V}$= .76, a value that is smaller than any individual’s discriminability. As shown in the right panel of Fig. 5, the concavity of the ROC function implies that the average of any two hit and false-alarm pairs coming from a single function (i.e., with the same discriminability) will always result in a pair that falls below that function. When evaluating a single experimental condition (e.g., syllogisms with believable conclusions), the distortions caused by aggregating heterogeneous participants can lead to an underestimation of discriminability.

Such underestimation of discriminability is especially pernicious when different experimental conditions are used (e.g., syllogisms with believable *versus* unbelievable conclusions, or reasoning under a fixed-time limit *versus* self-paced conditions, etc), as it can create spurious differences or mask real ones. For example, individuals might be better at discriminating syllogisms with believable conclusions than their unbelievable counterparts (cf., Guyote & Sternberg, 1981). But if the inter-individual variability in terms of the adopted response criteria is larger in the former than in the latter, then the resulting underestimation can mask the differences in discriminability. Alternatively, if discriminability is the same across two conditions, differences in terms of the inter-individual variability of response criteria can introduce spurious differences in the estimates obtained with the aggregate data. It is possible that some inconsistencies found in the literature (e.g., Dube et al., 2010; Klauer et al., 2000) are driven by this. For instance, Trippas et al., (2013), who also employed the SDT model, observed no effect of believability on discriminability *only* for participants of lower cognitive ability, with higher ability reasoners showing a more typical effect of beliefs on accuracy. This suggests that treating all participants as equivalent is perhaps not the best assumption.

## A hierarchical Bayesian meta-analytic approach

Fortunately, the problems associated with aggregation can be avoided by relying on hierarchical methods that take the heterogeneity at the participant and stimulus levels—logical structures in our case—into account (e.g., Baayen et al., 2008; Barr et al., 2013; Snijders & Bosker, 2012). Specifically, both participants and stimuli considered in the analyses are assumed to be random samples from higher group-level distributions, whose parameters are also estimated from the data. Note that when facing multiple studies, one can conceptualize each study as a random sample from a distribution of studies. Usually, each of these higher group-level distributions are assumed to follow a Gaussian distribution with some mean and variance. In the case of participant-level differences, the mean of this group-level distribution captures the average individual parameter value whereas the variance expresses the variability observed across participants. An analogous interpretation holds for the group-level distributions from which stimuli are assumed to originate.

Our hierarchical extension of SDT was implemented in a Bayesian framework (Gelman et al., 2013; Carpenter et al., 2017). In a Bayesian framework, the information one has regarding the parameters is represented by probability distributions. We begin by establishing *prior distributions* that capture our current state of ignorance. These prior distributions are then updated in light of the data using *Bayes’ theorem*, resulting in *posterior distributions* that reflect a new state of knowledge (for an overview of hierarchical Bayesian approaches, see Lee & Wagenmakers, 2013; Rouder & Jun, 2005). The estimation of posterior parameter distributions can be conducted using Markov chain Monte Carlo methods (for an introduction, see Robert & Casella, 2009). In the present work, we employed Hamiltonian Monte Carlo (e.g., Monnahan et al., 2016 and relied on weakly informative or non-informative priors that imposed minimal constraints on the values taken on by the parameters. These prior constraints are quickly overrun by the information present in the data.

The information captured by the posterior parameter distributions can be conveniently summarized by their respective means and 95% (highest-density) credible intervals. Each interval corresponds to the (smallest) region of values that include the true parameter value with probability .95. Moreover, the overall quality of a model can be checked by comparing the observed data with the predictions based on the model’s posterior parameter distributions (Gelman and Shalizi, 2013). If the observed data deviate substantially from the predictions then one can conclude that the model is failing to provide an adequate characterization.

### Hierarchical extension of signal-detection model

The contributions of participant and individual differences can be conveniently characterized in terms of a generalized linear model. For example, the probability that participant *p* will endorse a syllogism *s* could be described as

15 $$ P(\text{``valid''} | \text{Participant} = p, \text{Syllogism} = s) = {\Phi}(\bar{\mu} + \xi_{p} + \eta_{s}), $$

where $\bar {\mu }$ denotes the grand mean. Parameter $\xi _{p}$ corresponds to the *p* th participant’s displacement from that grand mean, whereas $\eta _{s}$ corresponds to the *s* th stimulus’s displacement. Displacements $\xi _{p}$ and $\eta _{s}$ are both assumed to come from zero-centered group-level distributions (for a similar approach, see Rouder et al., 2008). Based on this linear decomposition of participant and stimulus effects, the estimate of the overall probability of syllogisms being endorsed is given by ${\Phi }(\bar {\mu })$, the probability of participant *p* endorsing any syllogism corresponds to ${\Phi }(\bar {\mu } + \xi _{p})$, and the probability of syllogism *s* being endorsed by somebody is ${\Phi }(\bar {\mu } + \eta _{s})$.^5^ A hierarchical approach provides a compromise between the assumption that all participants and stimuli are effectively the same (as done when aggregating data) and the assumption that all participants and stimuli are unique (as done when analyzing data for each participant individually). Specifically, the assumption that both participants and stimuli come from group-level distributions implies the presence of differences between the participants/stimuli, but also the existence of similarities that should not be overlooked. The estimation of individual and group-level parameters are informed by each other—a principle known as *partial pooling*—leading to parameter estimates that are more reliable than what would be obtained via independent estimation from individual datasets (e.g., Ahn et al., 2011; Katahira, 2016; for a discussion, see Scheibehenne and Pachur, 2015).

As previously discussed, the SDT model characterizes individuals’ responses in terms of latent strength distributions defined with means $\mu $, standard deviations $\sigma $, and response criteria $\tau $. We will therefore introduce our hierarchical extension of SDT at the level of these parameters (Klauer, 2010; Rouder & Jun, 2005; Morey et al., 2008; Pratte & Rouder, 2011; Pratte et al., 2010). Because of the identifiability issues associated with SDT (see Footnote 3), we modeled believable and unbelievable syllogisms separately.

The probability of participant $p_{h}$ in experimental study *h* endorsing an invalid syllogism $s_{i}$ or valid syllogism $s_{v}$ are given by:

16 $$\begin{array}{@{}rcl@{}} \text{{FAR}}_{h,p_{h},s_{i}} = P(\text{\scriptsize{``valid''}} | \text{\scriptsize{Study = \(h\), Participant = \(p_{h}\), Syllogism = \(s_{i}\)}}) &=& {\Phi}\left(\frac{-\tau_{h,p_{h}} + \mu_{I,h,p_{h},s_{i}}}{\sigma_{I,h,p_{h},s_{i}}} \right), \end{array} $$

17 $$\begin{array}{@{}rcl@{}} \text{{HR}}_{h,p_{h},s_{v}} = P(\text{\scriptsize{``valid''}} | \text{\scriptsize{Study = \(h\), Participant = \(p_{h}\), Syllogism = \(s_{v}\)}}) &=& {\Phi}\left(\frac{-\tau_{h,p_{h}} + \mu_{V,h,p_{h},s_{v}}}{\sigma_{V,h,p_{h},s_{v}}}\right). \end{array} $$

Individual mean parameters $\mu _{I,h,p_{h},s_{i}}$ and $\mu _{V,h,p_{h},s_{i}}$ are established as a linear function of group-level means (${\bar \mu }$), and their respective experimental-study- (*χ*), participant- (*ξ*), and stimulus-level (*η*) deviations from those means:

18 $$\begin{array}{@{}rcl@{}} \mu_{I,h,p_{h},s_{i}} &=& {\bar\mu}_{I} + \chi^{\mu_{I}}_{h} + \xi^{\mu_{I}}_{p_{h}} + \eta^{\mu_{I}}_{s_{i}}, \end{array} $$

19 $$\begin{array}{@{}rcl@{}} \mu_{V,h,p_{h},s_{v}} &=& {\bar\mu}_{V} + \chi^{\mu_{V}}_{h} + \xi^{\mu_{V}}_{p_{h}} + \eta^{\mu_{V}}_{s_{v}}. \end{array} $$

Note the use of subscripts and superscripts (i.e., $\xi ^{\mu _{I}}_{p_{h}}$ does *not* mean “$\xi _{p_{h}}$ to the power of $\mu _{I}$”). For reference on the different parameters and sub/superscripts, see Table 2. Also, note that additional parameters could be easily added to the model, as is routinely done with predictor variables in multiple-regression models (we provide a demonstration of this in the general discussion). For example, Trippas et al. (2013, 2015) considered the relationship between individual differences variables such as cognitive ability and analytic cognitive style with SDT estimates of discriminability and response bias. The use of other predictor variables such as fMRI data have also been entertained (e.g., Roser et al., 2015).

**Table 2:** Description of hierarchical linear model parameters and super/subscripts

Parameter	Meaning
$\bar {\mu }$	Grand mean
*χ*	Study effect
*ξ*	Person effect
*η*	Item effect
Super/Subscript	Meaning
*V*/*I*	Valid/invalid
*h*	Study
*p*_*h*_	Participant in Study *h*
*s*	Syllogistic forms

A similar linear structure holds for the individual standard-deviation parameters $\sigma _{I,h,p_{h},s_{i}}$ and $\sigma _{V,h,p_{h},s_{i}}$, however it is implemented on a log scale:

20 $$\begin{array}{@{}rcl@{}} \sigma_{I,h,p_{h},s_{i}} &=& \exp\left( \log({\bar\sigma}_{I}) + \chi^{\sigma_{I}}_{h} + \xi^{\sigma_{I}}_{p_{h}} + \eta^{\sigma_{I}}_{s_{i}}\right), \end{array} $$

21 $$\begin{array}{@{}rcl@{}} \sigma_{V,h,p_{h},s_{v}} &=& \exp\left( \log({\bar\sigma}_{V}) + \chi^{\sigma_{V}}_{h} + \xi^{\sigma_{V}}_{p_{h}} + \eta^{\sigma_{V}}_{s_{v}}\right), \end{array} $$

where log() corresponds to the natural logarithm and exp() to the exponential function.

### Meta-analytic model

In terms of the meta-analytic model we implemented a variant of what is known as a *random-effects* or *random study-effects* meta-analysis (Borenstein et al., 2010; Whitehead, 2003). Note that the usage of the term ’random-effects’ in this context slightly differs from the other usage in this manuscript and simply means that our model allowed each individual study to have its own idiosyncratic effect and that we did not assume that all study had exactly the same overall effect. For the participant-level deviations $\xi ^{x}_{p_{h}}$ (where $x \in \{ \mu _{I}, \mu _{V}, \sigma _{I}, \sigma _{V} \}$) we assume they follow a normal distribution with mean 0 and study-specific variance $\sigma ^{2}_{\xi ^{x},h}$,

22 $$ \xi^{x}_{p_{h}} \sim \mathcal{N}(0, \sigma^{2}_{\xi^{x},h}), $$

where $\mathcal {N}$ corresponds to the distribution function of the normal or Gaussian distribution.^6^

For the study-specific deviations ${\chi ^{x}_{h}}$ we assumed the common random-effects meta-analytic model,

23 $$ {\chi^{x}_{h}} \sim \mathcal{N}(0, \sigma^{2}_{e,\xi^{x},h} + \upsilon^{2}), $$

where $\sigma ^{2}_{e,\xi ^{x},h}$ is the *within-study error variance*, $\sigma ^{2}_{e,\xi ^{x},h} = \frac {\sigma ^{2}_{\xi ^{x},h}}{N_{h}}$ (where $N_{h}$ is the number of participants in study *h*; $\sigma _{e,\xi ^{x},h}$ is also known as the standard-error), and $\upsilon ^{2}$ the *between-study error variance*.^7^ As can be seen from the previous two equations, the main difference between our meta-analysis based on the individual trial-level data and a traditional meta-analysis is that in our case the within-study error variance is estimated in the same step as all other parameters and not treated as observed data.

For ease of presentation, the formulas in the previous paragraph present a slight simplification of our actual model. For all displacement parameters, ${\chi ^{x}_{h}}$ (study-specific), $\xi ^{x}_{P_{h}}$ (participant-level), and ${\eta ^{x}_{s}}$ (stimulus/item-specific) we also estimated the correlation among the deviations across the different SDT parameters *x*. Thus, all displacements are actually assumed to come from a zero-centered multivariate Gaussian distributions with covariance matrices $\mathbf {{\Sigma }}_{S}$, $\mathbf {{\Sigma }}_{P}$, and $\mathbf {{\Sigma }}_{I}$, respectively (Klauer, 2010). For the covariance matrices $\mathbf {{\Sigma }}_{S}$ and $\mathbf {{\Sigma }}_{P}$ the standard deviations are as described in the previous paragraph and we additionally estimated one correlation matrix for each covariance matrix. For ${\eta ^{x}_{s}}$ we estimated one standard deviation for each *x* and one correlation matrix. The complete model is presented in the Appendix. The covariance matrices capture different dependencies that could be potentially found across participants’ parameter estimates. For instance, the participant-level covariance matrix $\mathbf {{\Sigma }}_{P}$ indicates how individual parameters, say $\mu _{V}$ and $\sigma _{V}$, covary across participants. The estimation of all these covariance matrices, which amount to a so-called “maximal random-effects structure” is strongly advised as it known to improve the generalizability and accuracy of the hierarchical model’s account of the data (Barr et al., 2013): Specifically, the hierarchical structure of the model’s parameters allows us to more safely make generalizations from our parameters of interest. For example, the group-level means (e.g., ${\bar \sigma }_{V}$) summarize the information that we have about the individuals, after factoring out their differences. These parameters allow us then to make general inferences regarding the population, such as whether $\sigma _{V}$ is systematically greater than $\sigma _{I}$, as currently claimed in literature (Dube et al., 2010; Heit & Rotello, 2014).

The extension of this model to the case of a *K*-point confidence-rating paradigm follows exactly what is already described in Eqs. 11 and 12, with the specification of $K-1$ ordered response criteria $\tau _{h,p_{h},k}$ per participant. The use of a different set of criteria per participant allows the model to capture different response styles that people often manifest (Tourangeau et al., 2000). As previously mentioned, it is customary to fix the parameters of the invalid-syllogism distributions, but in the present case we decided to instead fix $\tau _{h,p_{h},1}$ and $\tau _{h,p_{h},{K-1}}$ to 0 and 1, respectively. This restriction, which does not affect the ability of the model to account for ROC data, nor the interpretation of the parameters, implies that the mean and standard deviation parameters from all argument-strength distributions are freely estimated (for a similar approach, see Morey et al., 2008). The motivation behind the use of this particular set of parameter restrictions was that it provided a more convenient specification of the different sets of participant-, stimulus-, and group-level parameters and at the same time allowed for identical *prior distributions* (see below) for the two standard deviations $\sigma _{V}$ and $\sigma _{I}$, which are of interest here. Furthermore, we assumed that the remaining three response criteria per individual participant, $\tau _{h,p_{h},2}$ to $\tau _{h,p_{h},{K-2}}$, were each drawn from a separate group-level Gaussian distribution and then transformed on the unit scale using the cumulative distribution function of the standard Gaussian distribution. The sampling was performed such that the three to-be-estimated criteria per individual participant were ordered.^8^

In line with the literature (e.g., Dube et al., 2010; Trippas et al., 2013), we modeled the data for believable and unbelievable syllogisms separately using the same model. The reason for modeling these data separately is that SDT does not yield identifiable parameters (i.e., infinitely many sets of parameter values produce the exact same predictions; see Bamber & van Santen, 2000; Moran, 2016) when parameter restrictions are only applied on the parameters concerning one stimulus type (e.g., believable syllogisms) and everything else is left to be freely estimated (e.g., different response criteria for believable and unbelievable syllogisms). However, applying restrictions to each stimulus type while allowing criteria to vary freely between them is equivalent to fitting them separately (for detailed discussions; see Singmann, 2014; Wickens & Hirshman, 2000).

## Meta-analysis of extant ROC data

Our analysis differs from regular meta-analyses (e.g., Borenstein et al., 2010) in two important ways. First, we obtained the raw (i.e., participant- and trial-level) data and performed our meta-analysis on this non-aggregated data. This has the benefit that all variability estimates are obtained directly from the data and not inferred from other statistical indices. Second, our meta-analysis is performed using a fully *generative model*; it allows us to use the obtained parameter estimates to generate new synthetic data from for any part of the data corpus (e.g., for individual participants or studies). The data corpus and modeling scripts are available at: https://osf.io/8dfyv/.

The hierarchical Bayesian SDT model established here was fitted to a data corpus comprised of 22 studies, for a total of 993 participants. To the best of our knowledge, these datasets consist of all published and non-published studies on belief bias including ROC data for which individual- and item-level information is available. In the included datasets, (1) three-term categorical syllogisms were used as stimuli, (2) confidence ratings were collected on each trial, (3) data was available on the trial-level, and (4) information about the syllogistic structures was available for each trial. Over 80% (18/22) of the included studies were previously published. All of these studies involved participants evaluating the validity of believable and unbelievable syllogisms using a six-point confidence scale. Table 3 provides a description of these studies. An important aspect of these datasets is that they involve judgments obtained across a wide range of experimental conditions, in term of stimuli, instructions, response deadlines, stimulus-presentation conditions, among others. This diversity is particularly important when attempting to establish the robustness of any phenomenon, as it ensures that it is not circumscribed to a narrow set of conditions.

**Table 3:** Description of the data corpus

Study ID	N participants	N trials	Study
1	44	16	Trippas et al., (2013), Exp. 1, complex-syllogism condition
2	47	16	Trippas et al., (2013), Exp. 1, simple-syllogism condition
3	44	16	Trippas (2013), Exp. 6, no time limit
4	42	16	Trippas (2013), Exp. 6, 10s time limit
5	32	16	Trippas (2013), Exp. 7, deductive instructions
6	34	16	Trippas (2013), Exp. 7, weak instructions
7	36	16	Trippas et al., (2013), Exp. 2, 10s time limit, IQ
8	49	16	Trippas et al., (2013), Exp. 2, no time limit, IQ
9	45	8	Trippas (unpublished), complex-syllogisms, internal replication
10	38	16	Trippas et al., (2014), fluent-font condition
11	38	16	Trippas et al., (2014), disfluent-font condition
12	42	8	Nuobaraite (2013 dissertation), ego-depletion
13	24	8	Trippas (unpublished), complex-syllogisms, debias instructions
14	191	16	Trippas et al., (2015), individual differences
15	38	8	Dube et al., (2010), Exp. 2, complex-syllogisms
16	21	16	Dube et al., (2010), Exp. 3, conservative condition
17	24	16	Dube et al., (2010), Exp. 3, neutral condition
18	27	16	Dube et al., (2010), Exp. 3, liberal condition
19	45	8	Heit and Rotello (2014), Exp. 1, augmented instructions
20	44	8	Heit and Rotello (2014), Exp. 1, standard instructions
21	44	8	Heit and Rotello (2014), Exp. 2, conservative instructions
22	44	8	Heit and Rotello (2014), Exp. 2, standard instructions

*Note.* “N trials” gives the number of trials per participant and believability by validity cell (i.e., each participant responded to “N trials” times 4 syllogisms)

In terms of stimulus differences, we considered the different *forms* that syllogisms can take on. A categorical syllogism is an argument which consists of three terms, denoted here by A, B, and C, which are combined in two premises to produce a conclusion. The two terms which are present in the conclusion, A and C, are referred to as the *end terms*. The term which is present in each premise is referred to as the *middle term*, is denoted B. For example, in the “rose syllogism” given earlier, A = roses, B = petals, C = flowers. The two premises and conclusion each include one of four quantifiers: *Universal affirmative* (A; e.g., All A are B), *universal negative* (E; e.g., No A are B), *particular affirmative* (I; Some A are B), and *particular negative* (O; e.g., Some A are not B). The logical validity of a syllogistic structure is defined by its *mood*, its *figure*, and the *direction* of the terms in the conclusion. The mood is a description of which quantifiers occur in the syllogism. For instance, if the premises and the conclusion are preceded by the quantifiers “All”, “Some”, and “No”, respectively, then the syllogism’s mood is *AIE*. Given that a syllogism consists of three statements and that there are four possible quantifiers for each statement, there are 64 possible moods. The figure denotes how the terms in the conclusion are ordered. There are four possible figures: 1: (A-B; B-C), 2: (B-A; C-B), 3: (A-B, C-B), 4: (B-A; B-C).^9^ Finally, there are two possible conclusion directions: 1: (A-C) and 2: (C-A). Combining the 64 moods with the four figures and the two conclusion directions yields a total of 512 possible syllogisms, of which only 27 are logically valid (Evans et al., 1999). The combinations of form and figure in syllogisms can be conveniently coded by concatenating the two letters associated to the quantifiers of the premises, the number associated with the figure, the letter associated with the quantifier of the conclusion and the direction of the conclusion. The “rose syllogism” used earlier as an example would be coded as AA3_A2: both premises and conclusion start with the “All” quantifier, the syllogistic figure is 3, and the conclusion direction is 2—from C to A. A complete list of all the syllogistic figures used in the reanalyzed studies and their respective codes is included our supplemental material is hosted on the Open Science Framework (OSF). Specifically at: https://osf.io/8dfyv/.

### Results

**Fig. 6:**
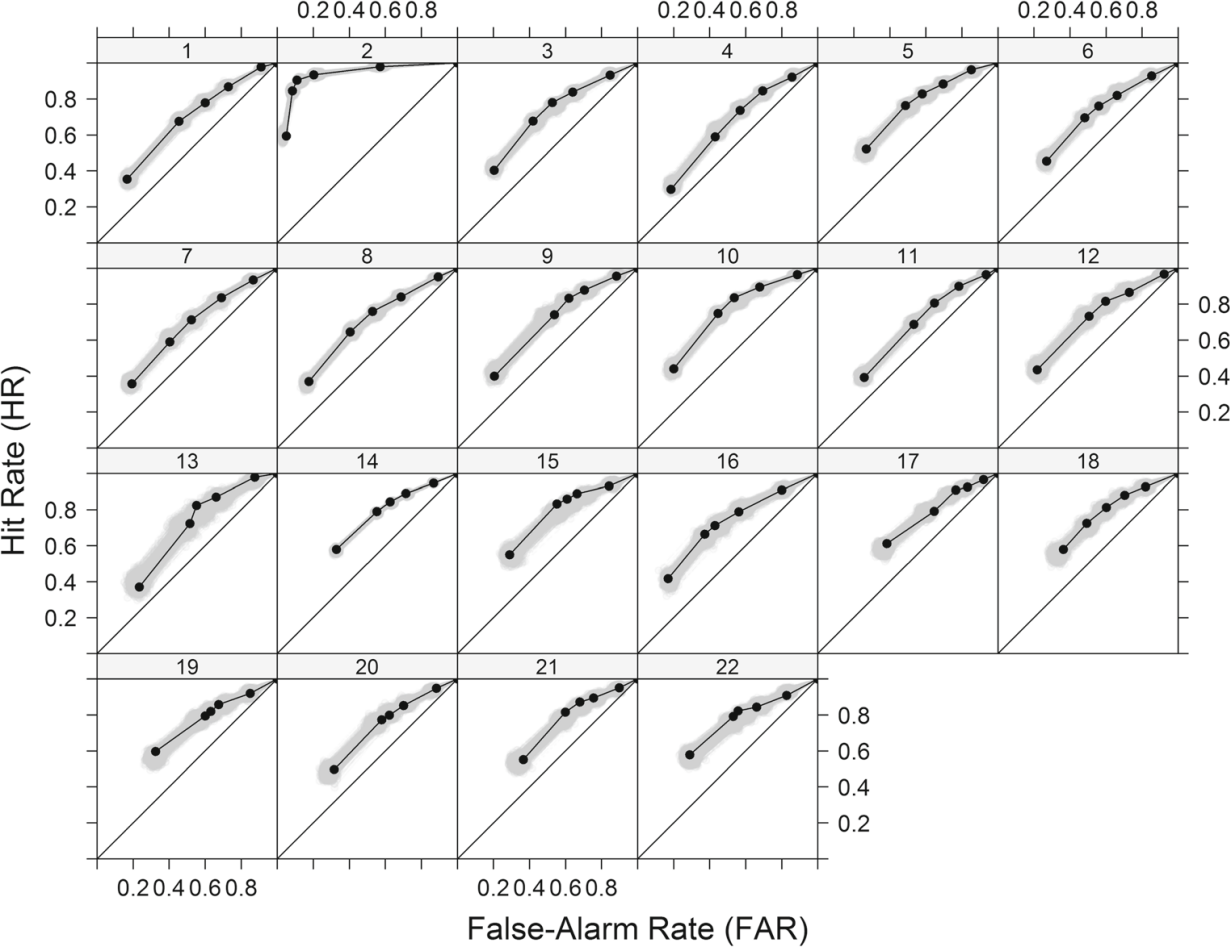
Believable-syllogism ROCs observed in each of the reanalyzed studies (for details, see Table 2). Note that these ROCs are based on the aggregated data. The *shaded regions* correspond to the hierarchical SDT model’s predictions based on its posterior parameter estimates

**Fig. 7:**
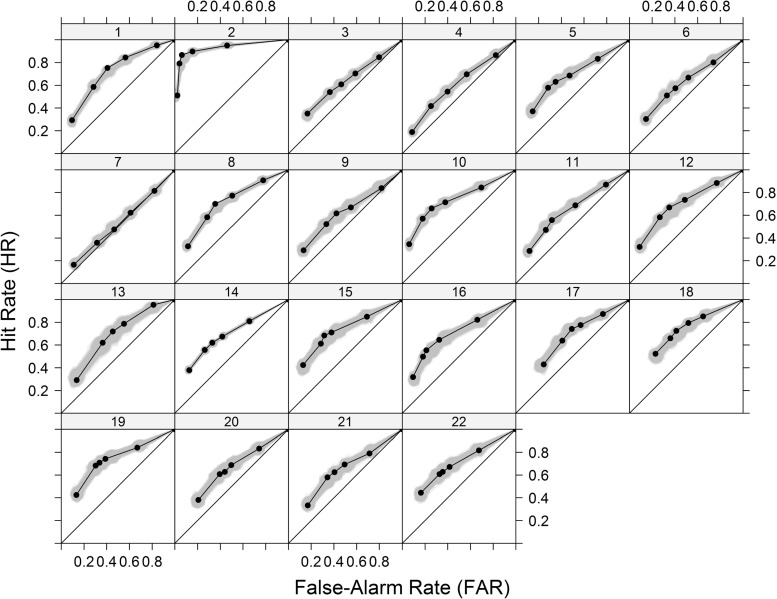
Unbelievable-syllogism ROCs observed in each of the reanalyzed studies (for details, see Table 2). Note that these ROCs are based on the aggregated data. The *shaded regions* correspond to the hierarchical SDT model’s predictions based on its posterior parameter estimates

We begin by evaluating the ability of the hierarchical model to fit the data. Specifically, we will evaluate its sufficiency (whether the model fits) and necessity (whether there is heterogeneity in stimuli and participants). With regards to the sufficiency of this hierarchical account, we implemented a model check by comparing the model predictions based on the model’s posterior parameter distributions and comparing it to the observed data (e.g., Gelman & Shalizi, 2013). Although SDT models for confidence-rating data are relatively flexible (Klauer, 2015), they cannot predict all possible data patterns in ROC space. This check allowed us to assess whether the model was able to describe the observed data sufficiently well. In this particular case, we generated one set of predictions based on each of the individual posterior-parameter distributions and subsequently aggregated them in order to compare with the ROCs obtained with the aggregate data. As can be seen in Figs. 6 and 7, the predictions based on the model’s posterior-parameter distributions are very similar to the ROCs observed across studies. This similarity strongly suggests that the model provides an adequate characterization of the data.

With regards to the necessity of a hierarchical account, we inspected the posterior estimates of the variability parameters of the participant- (${\bar \sigma _{\xi ^{x}}}$), stimulus- ($\sigma _{{\eta ^{x}_{s}}}$), and study-effects (*υ*) of the different SDT parameters. All of these variability parameters clearly deviated from zero (i.e., their 95% credible intervals do not include 0), indicating the presence of heterogeneity among participants, believable and unbelievable syllogisms, and studies. As discussed in detail by Smith and Batchelder (2008), the presence of such heterogeneity indicates the need for a hierarchical framework that does not rely on data aggregation.

**Fig. 8:**
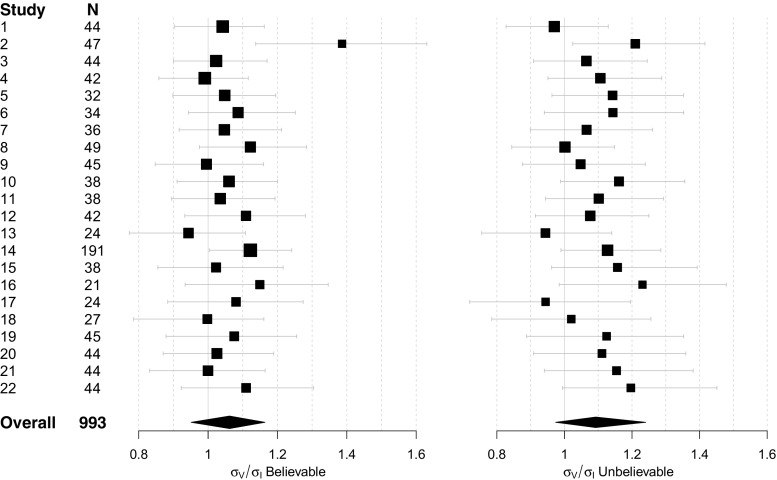
Posterior estimates of group-level $\frac {\sigma _{V}}{\sigma _{I}}$ observed in each study (*squares*; $\bar {\mu } + \chi ^{\sigma }$), and the posterior estimate obtained across studies (*diamonds*; $\bar {\mu }$ alone). The *bars* and the *width of the* *diamond* correspond to the 95% credible intervals. The *size of the squares* reflects the width of the credible intervals

The first question we posed was whether a simplified version of SDT could provide a sensible account of the data. As can be seen in Fig. 8, for both believable and unbelievable syllogisms, the posterior group-level estimates of $\frac {\sigma _{V}}{\sigma _{I}}$ were very close to 1 and their associated 95% credible intervals include values both above and below 1. Also, the posteriors were concentrated in a small range of values (see the diamonds in Fig. 8), reflecting the diagnostic value of the present data in terms of assessing ROC asymmetry. Overall, these results suggest that EVSDT, a simplified SDT model assuming that $\sigma _{V} = \sigma _{I}$, provides an adequate account of the data (Kruschke, 2015). Another way of framing this result is that data from almost 1000 participants were not sufficient to dismiss the EVSDT’s assumption that $\sigma _{V} = \sigma _{I}$. One exception to this pattern is Study 2, corresponding to the simple-syllogism condition of Trippas et al. (2013, Exp. 1), for which the posterior $\frac {\sigma _{V}}{\sigma _{I}}$ mean and 95% credibility interval are larger than 1. This result suggests that the ROC symmetry of the EVSDT model fails at extreme performance levels, as is the case for Study 2, where performance is close to ceiling.

In order to quantify the general degree of support for the EVSDT obtained from the posterior $\sigma _{V}$ and $\sigma _{I}$ estimates, we computed *Bayes factors* (BF; Kass & Raftery, 1995) that quantified the evidence in favor of EVSDT versus an unconstrained SDT model. In this specific case, the constrained EVSDT model was represented by the null hypothesis $\mathcal {H}_{0}$ stating that the group-level $\frac {\sigma _{V}}{\sigma _{I}}$ can take a small range of values, between .99 and 1.01, and an encompassing alternative hypothesis $\mathcal {H}_{A}$ that imposed no such constraint.^10^ In typical settings, the use of Bayes Factors requires the computation of marginal likelihoods for (at least) two models, which can be quite challenging (but see Gronau et al., 2017). But in this specific case in which the hypotheses considered consist of nested ranges of admissible parameter values (specifically, the range of $\frac {\sigma _{V}}{\sigma _{I}}$), Bayes Factors can be easily computed. As shown by Klugkist and Hoijtink (2007), the Bayes Factor for the two nested hypothesis corresponds to ratio of probabilities: The *posterior* probability that $.99 < \frac {\sigma _{V}}{\sigma _{I}} < 1.01$, and its *prior* counterpart. The obtained Bayes factors were 17.28 and 11.84 for believable and unbelievable syllogisms, which indicates that the posterior probability of $\frac {\sigma _{V}}{\sigma _{I}}$ values very close to 1 were 17 and 11 times greater after observing the data than before. According to the classification suggested by Vandekerckhove et al., (2015), this indicates strong support for $\mathcal {H}_{0}$.

**Fig. 9:**
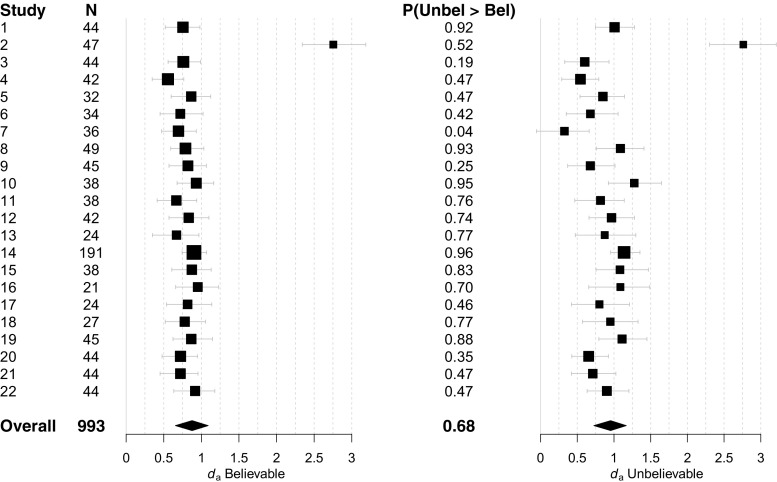
Forest plot with the posterior group-level estimates (and respective 95% credible intervals) of discriminability (*d*_*a*_) for believable and unbelievable syllogisms. The *size of the squares* reflects the width of the credible intervals. The probability P (Unbel > Bel) corresponds to the posterior probability that the group-level *d*_*a*_ estimate for unbelievable syllogisms is larger than for believable syllogisms

Let us now turn to our second question, whether there is a difference in discriminability for believable and unbelievable syllogisms. The group-level posterior $d_{a}$ estimates reported in Fig. 9 are virtually equivalent for believable and unbelievable syllogisms, with an almost complete overlap of their respective 95% credible intervals. This result indicates that the believability of conclusions does not have an impact on participants’ ability to discriminate between valid and invalid syllogisms, which is in line with Dube et al.’s (2010) findings. The present meta-analysis serves to dismiss any concerns that such a result could be due to aggregation biases or a handful of studies, and reiterates the challenge that it represents to the major theories proposed in the literature (Dube et al., 2010; Klauer et al., 2000). We quantified the strength of the evidence in favor of the null hypothesis that the differences in $d_{a}$ between believable and unbelievable syllogisms should take on a small range of values around 0 (values between $-$.01 and .01). We obtained a Bayes factor of 7.34, indicating substantial evidence in favor of $\mathcal {H}_{0}$.

**Fig. 10:**
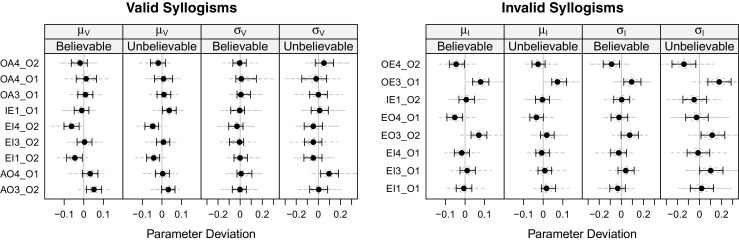
Posterior estimates (and respective 95% credible intervals) of the stimulus-level deviations (${\eta ^{x}_{s}}$) for the SDT parameters *x* concerning valid and invalid syllogisms (*s*_*v*_ or *s*_*i*_). The posterior estimates for the syllogisms used in study 2 are omitted here (see Foonote 5)

Figure 10 illustrates the posterior estimates of the stimulus-based differences (${\eta ^{x}_{s}}$) for believable and unbelievable syllogisms for the four SDT parameters for which we estimated stimulus effects.^11^ Overall, most differences are very close to zero; only for some forms did we find noteworthy deviations. These results indicate that the impact of the stimuli on the parameter estimates is small for most argument forms. However, note that the stimuli considered in 21 out of 22 studies came from only 16 syllogistic forms.

Figure 10 also allows us to compare our results to the meta-analysis of Khemlani and Johnson-Laird (2012). In contrast to the data considered here in which participants are presented with both premises and conclusion, they focused on data from the *conclusion generation task*. In this task participants are only provided with the premises and requested to create a possible conclusion or indicate that no conclusion follows. For the valid forms, our data are somewhat in line with their findings. The valid syllogisms that showed a clearly reduced discriminatibility with $\eta ^{\mu }_{s_{v}} < 0$, EI4_O2 and EI1_O2, also were among the most difficult according to Khemlani and Johnson-Laird (2012). Out of the 64 syllogistic forms their difficulty ranks (where 1 = easiest and 64 = most difficult) were 55 and 61, respectively. Interestingly, no such consistency can be found in the case of the invalid syllogisms: The two forms that clearly showed a reduced discriminability, OE4_O2 and EO4_O1, were relatively easy with ranks of 22 and 15, respectively. However, OE3_O1, which showed an increased discriminability in our study, $\eta ^{\mu }_{s_{i}} > 0$ was even slightly more difficult in the generation task with a rank of 26. These results reinforce the notion that the conclusion evaluation task and the conclusion generation task do not appear to involve the exact same cognitive processes. This would appear to carry additional implications for the mental models approach, beyond its seemingly faulty prediction of an effect of belief on reasoning, since much of the data used to develop the mental models theory of conclusion evaluation tasks was obtained using the production task. Furthermore, the model’s assumption that evaluation is implicit production is also questioned by these results.

## Validity checks

In this section, we will discuss different ways in which we attempted to corroborate our results. We relied on different approaches such as prior sensitivity analysis, assessing the impact of aggregation biases, and parameter recovery simulations. As discussed in detail below, all of the results support the conclusions from our meta-analysis.

### Prior sensitivity analysis

We begin by evaluating how strongly our results depend on the particular prior distributions. To this end, we fitted an additional model with alternative priors. More specifically, we specified markedly wider prior distributions for most parameters (e.g., the prior for both ${\bar \mu }$ in the original model was a Cauchy distribution with location = .5 and scale = 4; in the alternative model it was a Cauchy distribution with location = .5 and scale = 10). As shown in Table 4, the model with the alternative priors produced essentially the same results, both in terms of posterior distributions as well as in terms of Bayes Factors.

**Table 4:** Validity Checks

Model	Parameter / Derived Measure			
	$\bar {\mu }_{V}$ Believable	$\bar {\mu }_{I}$ Believable	$\bar {\mu }_{V}$ Unbelievable	$\bar {\mu }_{I}$ Unbelievable
Original	1.05 [.97, 1.14]	.62 [.54, .71]	.79 [.72, .86]	.32 [.25, .38]
Alternative	1.05 [.97, 1.13]	.62 [.54, .70]	.78 [.72, .85]	.32 [.25, .39]
No ${\eta ^{x}_{s}}$	1.05 [.98, 1.14]	.63 [.56, .71]	.78 [.72, .85]	.33 [.27, .39]
No $\xi ^{x}$, no ${\eta ^{x}_{s}}$	.98 [.92, 1.05]	.61 [.53, .68]	.76 [.69, .82]	.34 [.29, .40]
$\frac {\bar {\sigma }_{V}}{\bar {\sigma }_{I}} = 1.50$	1.05 [.98, 1.12]	.61 [.52, .70]	.77 [.70, .84]	.32 [.27, .38]
	$\bar {\sigma }_{V}$ Believable	$\bar {\sigma }_{I}$ Believable	$\bar {\sigma }_{V}$ Unbelievable	$\bar {\sigma }_{I}$ Unbelievable
Original	.50 [.46, .55]	.47 [.43, .53]	.51 [.45, .58]	.47 [.41, .54]
Alternative	.50 [.46, .54]	.47 [.43, .52]	.51 [.45, .57]	.47 [.41, .53]
No ${\eta ^{x}_{s}}$	.50 [.46, .55]	.48 [.44, .53]	.52 [.46, .58]	.49 [.44, .55]
No $\xi ^{x}$, no ${\eta ^{x}_{s}}$	.58 [.53, .62]	.56 [.52, .60]	.68 [.61, .76]	.56 [.52, .61]
$\frac {\bar {\sigma }_{V}}{\bar {\sigma }_{I}} = 1.50$	.70 [.65, .76]	.48 [.43, .52]	.69 [.62, .76]	.47 [.41, .53]
	$\frac {\bar {\sigma }_{V}}{\bar {\sigma }_{I}}$ Believable	$\frac {\bar {\sigma }_{V}}{\bar {\sigma }_{I}}$ Unbelievable	$d_{a}$ Believable	$d_{a}$ Unbelievable
Original	1.06 [.95, 1.17]	1.09 [.96, 1.23]	.62 [.46, .78]	.67 [.52, .83]
Alternative	1.06 [.95, 1.17]	1.09 [.95, 1.23]	.63 [.47, .78]	.67 [.52, .83]
No ${\eta ^{x}_{s}}$	1.05 [.97, 1.12]	1.05 [.98, 1.13]	.60 [.47, .74]	.64 [.50, .78]
No $\xi ^{x}$, no ${\eta ^{x}_{s}}$	1.03 [.99, 1.08]	1.21 [1.14, 1.28]	.46 [.35, .58]	.47 [.35, .59]
$\frac {\bar {\sigma }_{V}}{\bar {\sigma }_{I}} = 1.50$	1.48 [1.35, 1.61]	1.45 [1.30, 1.62]	.51 [.39, .64]	.54 [.42, .67]

*Note.* Values in [ ] correspond to the 95% credible intervals of the posterior distributions. The “Original” model is referred to throughout the results section. The “Alternative” model has the same structure but a different prior distribution specification. The “No ${\eta ^{x}_{s}}$” model has no stimulus effect (i.e., data aggregated within participants) and the “No $\xi ^{x}$, no ${\eta ^{x}_{s}}$” has neither participant nor study effect (i.e., data aggregated within studies), both models are otherwise identical to the “Original” model. The “$\frac {\bar {\sigma }_{V}}{\bar {\sigma }_{I}} = 1.50$” model uses the same priors as the “Original” model, but is fitted to data generated from the parameters of the “Original” model with the sole difference that $\bar {\sigma }_{V} = 1.5\times \bar {\sigma }_{I}$

### Effects of data aggregation

In the first part of this report, we provided several theoretical and simulation-based arguments illustrating why data aggregation can lead to biased conclusions. We now address this question empirically by reanalyzing our data corpus with models in which we purposefully omitted potential sources of variability, such as stimuli or participants. Given these concerns, it is interesting to see the extent to which aggregation actually affects results. For example, Pratte et al., (2010) found, in the context of recognition memory, that aggregation biases did not ultimately affect the observation of asymmetric ROCs. This outcome suggests that data aggregation may not be problematic as typically portrayed. Does a similar situation hold here? To find out, we checked whether we found evidence *against* the EVSDT when aggregating across the different sources of variability. In the first of those reanalysis we did not include stimulus-specific differences and aggregated the data within participants (model “no ${\eta ^{x}_{s}}$”). This model resulted in parameter estimates that were nearly identical to those of the original model (see Table 4), in line with the earlier observation that the stimulus-specific effects were rather modest. However, the confidence bands for $\frac {\sigma _{V}}{\sigma _{I}}$ were markedly narrower when compared with the original model. This result indicates that data aggregation can affect parameter estimates by attributing them an unwarranted degree of certainty.

In the second reanalysis we only analyzed the data aggregated on the study level, ignoring both a stimulus-specific effect as well as a participant-specific effect (model “no $\xi ^{x}$, no ${\eta ^{x}_{s}}$”).^12^ This is the analysis most often performed in previous work on reasoning ROCs (e.g., Dube et al., 2010, although we employed a Bayesian approach here as well). For this model (see Table 4) we now find rather strong differences relative to the other variants. Furthermore, for the unbelievable syllogisms we now find clear evidence against EVSDT with $\frac {\sigma _{V}}{\sigma _{I}} = 1.21$ [1.14, 1.28].

Taken together, these reanalyses reinforce two important points. First, ignoring random variability that is part of the data can lead to aggregation artifacts such as evidence for the unconstrained SDT model although the simpler EVSDT model is in fact more likely to be the data-generating model. This also explains why earlier studies found such evidence. Second, even in cases in which the random variability does not distort the parameter estimates in dramatic ways it can still lead to estimates purporting a precision that is not actually warranted by the data. Both of these results reinforce the dictum of Barr et al., (2013): always employ the *maximal random-effects structure justified by the design* (see also Schielzeth & Forstmeier, 2009).

### Parameter-recovery simulation

In the second step we evaluated our ability to recover model parameters. The idea here is that we should be confident about our results only if we can demonstrate that our hierarchical Bayesian SDT model can recover the data-generating parameters. Specifically, we evaluated our ability to recover parameters when the generated data are *not* in line with the EVSDT, with $\frac {\sigma _{V}}{\sigma _{I}} = 1.50$ (a value that is in line with the estimates obtained in other domains; e.g., Starns et al., 2012). In this simulation, we relied on the parameter estimates obtained from the present meta-analysis in order to have realistic individual parameter values. Specifically, we generated one data set identical in size to the original data from the parameter estimates obtained from the original model with the sole difference that $\sigma _{V} = 1.5 \times \sigma _{I}$ and then used the original model to fit the data. We were able to recover parameter estimates, which were at odds with the EVSDT. Table 4 reports the results obtained from the group-level estimates, which are close to the data-generating parameters (compared with the parameter estimates obtained in the meta-analysis, also reported in Table 4), reinforcing our trust in the present results. These results also dismiss the concern that the ROC datasets have limited diagnostic value, as some of them appear to only cover some of the possible range of hits and false-alarm values. If the data were not diagnostic for detecting asymmetries, then the present recovery of the $\frac {\sigma _{V}}{\sigma _{I}}$ ratio would have not been expected.

Having established that our meta-analytic results are trustworthy and the data diagnostic, we now present that data from an experiment featuring a critical test of our main novel finding: ROC symmetry.

## A critical test of ROC symmetry

So far, we have estimated the shape of ROC data on the sole basis of participants’ confidence-rating judgments. An exclusive reliance on such data may be problematic: it is possible that researchers relying on a single type of data can fall victim to mono-operation biases (Shadish et al., 2002, Chap. 3). Indeed, there is the question of whether ROCs obtained with confidence ratings match ROCs obtained with other methods (e.g., response-bias or payoff manipulations; see Klauer & Kellen, 2010; Klauer, 2011; Kellen et al., 2013). Furthermore, it has been suggested that the mere act of collecting confidence ratings may critically alter the decision process (Malmberg, 2002). Ideally, one seek converging evidence for the meta-analytic results supporting ROC symmetry with novel experimental data coming from alternative experimental paradigms to provide converging evidence.

One approach would consist of collecting ROC data without relying on confidence-rating judgments but instead use response bias or payoff manipulations. This approach is in many ways problematic: on a practical level, participants tend to be quite conservative when it comes to shifting their response criteria across response-bias conditions, leading to ROC points that are too close to evaluate the overall shape of the ROC (e.g., Dube & Rotello, 2012). On a theoretical level, there is a risk that individuals do not maintain the same level of discriminability across response bias conditions, compromising ROC analysis (which assumes that discriminability remains constant; see Balakrishnan, 1999; Bröder & Malejka, 2016; Van Zandt, 2000).

In order to sidestep these issues, we conducted a critical test of ROC symmetry that capitalizes on an overlooked property of SDT that was originally established by Iverson and Bamber (1997). In a result known as the *Generalized Area Theorem*, Iverson and Bamber showed that the ROC function of a decision maker can be characterized by his/her performance across different *M*-alternative forced-choice trials in which one tries to identify the target stimulus (e.g., the valid syllogism) among M-1 lure stimuli (e.g., invalid syllogisms). Specifically, the proportion of correct responses in a *M*-alternative forced-choice (*M*-AFC) task corresponds to the Mth moment of the ROC function (for a detailed discussion, see Kellen, 2018). This result is completely non-parametric as it does not hinge on the latent distributions taking on a specific parametric form (i.e., the distributions do not have to be Gaussian). The Area Theorem popularized by Green (see Green & Moses, 1966), which states that the proportion of correct responses in 2-AFC task corresponds to the area under the ROC function (i.e., the function’s expected value or first moment), is an instance of the Generalized Area Theorem.

Iverson and Bamber (1997) showed that the generalized area theorem also enabled ROC symmetry to be tested on the basis of *M*-alternative forced-choice judgments: consider a complementary forced-choice task, designed here as *M*-*C* AFC, in which the decision maker is requested to identify the lure stimulus among $M-1$ target stimuli. For example, in a 4-AFC task the decision maker is presented with three invalid syllogisms and one valid syllogism and has to pick the valid one, whereas in the 4-*C* AFC the decision maker is presented with one invalid syllogism and three valid ones and has to pick the invalid one. It can be shown that an ROC function is symmetric (Killeen & Taylor, 2004) if and only if, for all *M*, the proportions of correct judgments in *M*-AFC and *M*-CAFC tasks are the same (for details, see Iverson & Bamber, 1997).

### Method

#### Participants

We collected data in an online web-based study advertised on Amazon Mechanical Turk with a pre-determined stopping rule of 125 participants. Participants were paid 1.25 USD for their participation, which took approximately 20 min. Ethical approval for the study was granted by the Office of Research Ethics at the University of Waterloo, Canada.

#### Procedure

Given the possibility for online data to be more noisy than the equivalent lab data, we built in a number of checks to ensure the data quality was sufficiently high. Upon agreeing to participate in the experiment, an informed consent page was presented. After providing informed consent by clicking a button saying “I Agree”, the following instructions were presented:

In this experiment we are interested in yourability to reason according to the rules oflogic. You will be presented with a numberof reasoning puzzles (or "arguments") whichconsist of two premises and a conclusion.Some of these puzzles will have a logicallyVALID conclusion, others will have alogically INVALID conclusion(explained below). Your task is todiscriminate between logically VALID andINVALID arguments.ᅟAn argument is VALID if its conclusionnecessarily follows from thepremises, assuming the premises are true.For instance:Premise 1: All A are BPremise 2: All B are CConclusion: All A are CThe conclusion (All A are C) is logicallyVALID because if you assume it is true thatAll A are B and that all B are C, then itnecessarily follows that all A are C.By contrast, the conclusion All C are A islogically INVALID, because assuming thepremises are true, this does not necessarilyfollow. The entire experiment consists of 24trials, divided in two blocks of 12. This isa control question. If you have read theseinstructions carefully, please type in theword "reasoning" below where it says"respond here". >> Respond here: ________

Participants who did not correctly answer the control question within five attempts were not allowed to participate in the study (they were still paid). Participants who correctly answered the control question were presented with the next set of instructions, which read:

In the first part of the study, you will bepresented with up to four of these logicalreasoning problems at the same time. Atleast one of these arguments will always belogically VALID [INVALID], and the remainingwill be logically INVALID [VALID]. Your taskis to select the box which you thinkcontains the logically VALID [INVALID]argument by clicking the box containing thatargument. To start with the experiment,please type in the field below which box youshould select (in lowercase):>> The box containing the _______ argument.

We tested the symmetry assumption in syllogistic reasoning using *M*-AFC and a *M*-CAFC tasks for *M* = 2, 3, and 4. The participants were given 24 forced-choice trials containing two, three, or four abstract syllogisms side-by-side (*M* was manipulated within participants), either under instructions to choose the valid argument (*M*-AFC task) or under instructions to choose the invalid argument (*M*-CAFC task), in a blocked and counterbalanced design (four trials per cell of the design). In contrast with the data used in the meta-analysis, we did not manipulate the believability of the conclusions (for an application of 2-AFC to the study of belief bias, see Trippas et al., 2014).

### Results

The individual choice data were analyzed with a hierarchical Bayesian probit-regression model that included the main effects of “number of alternatives” (two, three, or four) and “choice focus” (choose target or lure item), as well as their interaction. Weakly-informative priors were set for all effects, with a normal distribution with mean 0 and standard deviation 4 and 16 being assigned to the intercept and slope coefficients, respectively. Here, our interest lies in whether there is a robust effect of “choice focus” (if there is, then the ROC is asymmetrical). When attempting to choose the invalid syllogism, the group-level estimates of correct-choice probabilities were .60 [.55, .65], .43 [.38, .49], and .34 [.29, .39] for *M* $=$ 2, 3, and 4, respectively. When attempting to choose the valid syllogism, the analogous estimates were .64 [.58, .69], .43 [.38, .49], and .38 [.32, .43]. Both sets of estimates appear to be similar, in line with the notion that ROCs are symmetrical. Indeed, the main effect of “choice focus” was merely -.03 [-.08, .02]. We computed a Bayes factor that quantified the relative evidence in favor of the null hypothesis that the latter effect is zero (in contrast with the alternative hypothesis that it is not zero). The obtained value was 69.72, which indicates very strong evidence in favor of the null hypothesis. Overall, the results show that our argument for ROC symmetry does not exclusively hinge on data from confidence-rating paradigms, dismissing the notion of a mono-operation bias in our meta-analytic results. More importantly, they provide converging evidence using a novel paradigm, suggesting that the equal variance SDT model is an appropriate model for belief bias in syllogistic reasoning. We discuss the implications of this experiment and the meta-analysis in next section.

## Discussion

We can extract two take-home messages from the meta-analysis and critical experimental test: (1) judgments in syllogistic reasoning seem to be well accounted by the EVSDT model, which in turn is equivalent to a probit-regression model. (2) Individuals show the same discriminability between valid and invalid syllogisms for believable and unbelievable syllogisms. These two results have serious implications on an empirical, methodological, and theoretical level. On an empirical level, the fact that the EVSDT model can be applied to binary judgments means that one can safely revisit a large body of work, as long as participant- and stimulus-level differences are taken into account. EVSDT appears to fail when performance is at ceiling (e.g., Study 2), but such performance levels are very far from what is typically observed in syllogistic reasoning studies, in which many errors are made, and the focus is placed on the nature of such errors (e.g., Khemlani & Johnson-Laird, 2012). Altogether, the routine collection of confidence ratings does not seem *necessary* for the appropriate measurement of belief bias–though we hasten to add that doing so could certainly be of interest from a meta-cognitive perspective (Ackerman & Thompson, 2015; Thompson et al., 2011). Finally, on a theoretical level, the results seem to corroborate Dube et al., (2010) in the sense that the lack of an effect of believability on discriminability is at odds with nearly all extant theories of syllogistic reasoning. At least as long as one does not take further individual characteristics into account as done below.

Meta-analyses are typically conducted with the goal of obtaining a “final word” on a given subject. In the present case, we reject such a view. Instead, we believe that our results should be framed as establishing a new starting point for research on syllogistic reasoning. This starting point involves the incorporation of some important facts: The exact way in which we relate data and theoretical constructs matters. Differences across studies, participants, and stimuli matter. That ignoring any of the latter should be seen as dangerous and misinformative. Based on this standpoint, we will dedicate the remainder of this paper to the discussion of how one can build upon the present work and develop better and more comprehensive characterizations of deductive reasoning.

### Relating individual reasoning abilities and theories of belief bias

The hierarchical Bayesian SDT approach used here incorporates many state-of-the-art methods that deal with different confounds such as the heterogeneity found at the level of participants and stimuli. At this point, we do not see how one could significantly improve upon the present approach based on the *available data alone*. But despite the merits of such an approach, we believe that some important limitations still need to be addressed. Chief among them is the fact that although the model can capture individual differences, it is *completely silent* regarding any of the factors that underlie them. Given the considerable body of work showing that different groups of individuals attempt to reason in qualitatively distinct ways (e.g., Stupple et al., 2011), it is extremely likely that the inclusion of *additional* individual-level information might reveal new patterns and insights that have so far only been investigated using the SDT model applied to aggregate data (Trippas et al., 2013, 2015, 2014. In particular, these studies suggest that the addition of idiographic information might lead to a *reframing* of current theories of syllogistic reasoning rather than the strong dismissal suggested by the lack of an effect of believability on reasoning accuracy reported here.

Let us entertain the hypothesis that a sample of participants is comprised of elements from two groups, *M* and *T*: Group *M* consists of people who reason in accordance to the *mental-model theory* (Oakhill et al., 1989) given their stronger tendency to manifest an analytic cognitive style (e.g., Pennycook et al., 2015). By reasoning in accordance to the principles of the mental-model theory, they will typically reason better for unbelievable syllogisms, as these conclusions will trigger a search for counterexamples. Group *T* is made up of participants, who by having a lower tendency to manifest an analytic cognitive style, tend to reason in accordance to the *transitive-chain theory* (Guyote and Sternberg, 1981). These people are then expected to reason worse for unbelievable syllogisms than for believable ones, as the unbelievable contents are more challenging to manipulate mentally. Analyzing data from such an experiment under the assumption that everybody amounts to some variation of the same reasoning strategy is likely to yield the incorrect conclusion that beliefs do not affect discriminability (as the differences in discriminability found in both groups can cancel each other out), in line with Dube et al.’s (2010) account.

This example can be made more concrete by reanalyzing Study 14 (Trippas et al., 2015), a large sample study (*N* = 191) in which additional individual information was available for 182 participants in the form of the Cognitive Reflection Test (CRT; Frederick, 2005). The CRT is a test which consists of three simple but surprisingly tricky problems which have been shown to capture individual differences in analytic cognitive style—that is, the degree to which a participant tends to engage in analytical thought (Pennycook et al., 2016; Toplak et al., 2011). As an example, consider the following question from the CRT (the *widgets* problem): “if a factory with 100 workers produces 100 widgets in 100 days, how many days would it take for 5 workers to produce 5 widgets?”. The intuitive response (based on a matching-heuristic) is “5 days”. However, the correct response is in fact “100 days”—after all, the problem premise entails that it takes 1 worker 100 days to produce 1 widget. We classified people who responded correctly to at least one problem as part of the “analytic” group (*N* = 111). People who responded incorrectly to all three problems were classified as part of the “intuitive” group (*N* = 71).

We reanalyzed the binary endorsement rates for this study using the EVSDT/probit-regression model that was validated by the SDT analysis. But instead of only considering participant- and stimulus-level differences as done before, we also included those participants’ respective CRT classifications. The equation of this hierarchical Bayesian probit-regression model is

25 $$\begin{array}{@{}rcl@{}} P(\text{``valid''} | \text{participant}=p, \text{Syllogism} = s_{L}, L, B, C) &=& {\Phi} (\beta_{0,p} + L\beta_{L,p} + B\beta_{B,p} + C\beta_{C,p} \\ && + LB\beta_{LB,p} + LC\beta_{\text{LC},p} + BC\beta_{BC,p}\\ && + LBC\beta_{LBC,i} + \beta_{s_{L}}). \end{array} $$

This model includes parameters capturing the main effects of Logic (L), Belief (B), and CRT classification (C), but also their two- and three-way interactions (all factors are again-1/1 coded). These parameters are allowed to vary across participants, allowing for individual differences to be taken into account. Moreover, $\beta _{s_{L}}$ captures stimulus differences among valid and invalid syllogisms (additional details and code to implement this model are presented in Appendix B). The results reveal, among a host of less-surprising effects, a credible group-level three-way Logic $\times $ Belief $\times $ CRT-classification interaction $\beta _{LBC}$ = -.07 [-.10, -.03], suggesting that the Logic $\times $ Belief interaction is moderated by individual differences in analytic cognitive style as measured by the CRT. Specifically, this finding suggests that participants classified as “analytic” reasoners tend to perform better when evaluating unbelievable syllogisms (group-level $d^{\prime }$ = 1.38 [1.20, 1.56]) than believable ones (group-level $d^{\prime }$ = .91 [.81, 1.02]). For participants classified as “intuitive” reasoners, the effect is smaller in magnitude and, if anything, reversed, with group-level $d_{unbelievable}^{\prime }$ = .49 [.29, .69] and $d_{believable}^{\prime }$ = .55 [.44, .66]. This pattern is in line with the scenario described above, with better reasoners behaving in accord with mental-models theory, and worse reasoners with transitive-chain theory. Although far from clear cut, these results demonstrate the added value coming from the inclusion of individual covariates, pointing researchers towards an individual-differences approach already adopted in domains such as emotion research and psychometrics (Rijmen et al., 2003).

In our view, the statistical model used in this reanalysis should be considered as the new standard in analyzing endorsement rates in syllogistic reasoning: (1) It respects the nature of the data (categorical responses), (2) it is based on a validated EVSDT model, (3) it takes into account the heterogeneity found across participants and stimuli, and (4) it can be easily extended to include additional covariates. This model can also be conveniently implemented by researchers. Here, we relied on the R package rstanarm (Gabry & Goodrich, 2016). Appendix B provides details on how the model is specified (a complete script along with data can be found in our supplemental material is hosted on the Open Science Framework (OSF). Specifically at: https://osf.io/8dfyv/).

### Beyond pure (SDT) model and single-task approaches

Throughout this manuscript, we exclusively relied on the SDT model framework. However, this is not the only approach that could be successfully adopted. For instance, many researchers often rely on *discrete-state models* based on *multinomial processing trees* (for an overview, see Batchelder & Riefer, 1999; Erdfelder et al., 2009). Instead of describing responses in terms of continuous latent representations (e.g., distributions on an argument-strength scale), these assume that responses are produced by a finite mixture of discrete cognitive states that are entered probabilistically. For example, Klauer et al. (2000) considered a discrete-state model in which the true logical status of a valid syllogism is detected with a certain probability (e.g., probability $D_{v}$), a state in which a correct judgment was invariably made. When the logical status of a valid syllogism is not detected (e.g., with probability $1-D_{v}$), the model assumes that individuals simply guess whether the syllogism is valid or invalid (with probabilities *g* and $1-g$, respectively). By testing detection probabilities and guessing biases across different types of syllogisms and experimental conditions, Klauer et al. were able to establish a testbed for the predictions of many different models of syllogistic reasoning.

Several successful discrete-state approaches can be found in the reasoning literature, outside of the context of the beliefbias effect discussed here (Böckenholt, 2012b; Campitelli & Gerrans, 2014; Oberauer, 2006; Oberauer et al., 2006; Klauer et al., 2007; Krauth, 1982). For example, Klauer et al., (2007) developed a discrete-state model for the classic Wason selection task (Wason, 1966), which requires participants to decide which of four cards needs to be flipped in order to test a given rule (“If there is an A on the letter side, then there is a 3 on the number side”). This discrete-state model establishes how the observed responses (among the 16 possible combinations of card turns) can result from different interpretations of the rule (e.g., conditional versus biconditional interpretation), the types of inferences considered (forward versus backward), and their perceived sufficiency or necessity (see also Oberauer, 2006). Another example worth mentioning is the *cognitive-miser model* originally proposed by Böckenholt (2012b) and further developed by Campitelli and Gerrans (2014). This model, which is used to characterize responses from an extended version of the CRT, allows for the estimation of thinking dispositions and mathematical abilities by establishing parameters reflecting the probability of successful response inhibition and deliberative processing being engaged.

There is a decade-long debate among SDT and discrete-state modelers on the relative merits of the two approaches in several psychological domains (Batchelder et al.,, 1994; Dube & Rotello, 2012; Dube et al.,, 2010, 2012; Kellen & Klauer, 2011; Kellen, 2014; Kellen et al.,, 2013, 2015; Kinchla, 1994); for reviews, see Pazzaglia et al., (2013), Batchelder and Alexander (2013), and Dube et al., (2013).^13^ From this heated debate, two constructive points are often overlooked: First, there is some consensus that the two modeling approaches seem to be particularly successful in certain types of domains and paradigms. For instance, discrete-state approaches allow for a more clear separation between mental states and their mapping onto observed responses, which has enabled researchers to develop a wide range of methods to account for individual differences in response styles (see Böckenholt, 2012a; Klauer & Kellen, 2010). Second, the two modeling approaches can be conveniently integrated in order to create hybrid models that simultaneously account for different kinds of data. As pointed out by Klauer and Kellen (2011), the parameters expressing the probability of different discrete states being entered can be easily specified as a function of continuous distributions like the ones postulated by SDT (see also Klauer, 2010).

A combination of these modeling approaches, particularly when done in a hierarchical Bayesian fashion, opens very promising avenues of research. For instance, one can integrate the cognitive-miser and SDT models in order to further explore the relationships between different reasoning theories and the belief-bias effect. Moreover, one can develop hybrid models that bridge the gap between different types of data that are relevant for theories of syllogistic reasoning. For example, Khemlani and Johnson-Laird (2012) tested a large set of models of syllogistic reasoning using data from a conclusion generation task in which participants attempted to produce a conclusion from a given pair of premises. The categorical data coming from this task (note that participants can produce many types of conclusions) could be conveniently modeled by means of discrete states. It would be interesting to try to link the parameters describing the probabilities of such states being entered with the argument-strength distributions that underlie the SDT modeling of endorsement rates. The joint modeling of both tasks simultaneously could help researchers to better understand the general and task-specific aspects of the data (e.g., the previously discussed fact that the difficulty of invalid syllogisms appears to differ between tasks). These joint-modeling efforts seem particularly important when considering the recent efforts to integrate different reasoning abilities within a single framework (e.g., Stanovich et al., 2016; Thompson, 2000).

### Playing a more ambitious game

Allen Newell famously stated that one cannot hope to play “20 Questions” with Nature and win. Khemlani and Johnson-Laird (2012) faced such a humbling situation when failing to find a theory that successfully accounted for 64 different syllogistic forms. The difficulties associated with describing the wide range of syllogisms available has led many researchers to focus their efforts on a few cases only. Despite its practical appeal, this strategy has led to the present case in which the 22 reanalyzed datasets pretty much focused on 17 syllogistic forms. Another advantage of the hierarchical Bayesian SDT approach advocated here is that it allows for a characterization of the different syllogistic forms without any form of aggregation (note that Khemlani and Johnson-Laird (2012), relied on aggregate data) that can later guide us towards more comprehensive theories of syllogistic reasoning. In fact, one could in principle connect the SDT model with more fine-grained computational theories by constraining the parameters of the former to be a function of the mechanisms of the latter (for examples in the context of recognition memory, see Brandt, 2007; Osth & Dennis, 2015).

Last but not least, future work should attempt to go beyond acceptance rates and incorporate the time take taken for making these judgments. For instance, one can rely on the *drift-diffusion model* (e.g., Ratcliff & Rouder, 1998), which can be seen as a dynamic extension of the SDT model used here. However, note that other options are available, including the use of a dynamic discrete-state model approach (e.g., Klauer, 2018). Although response times have not played a significant role in this literature, they nevertheless introduce important theoretical constraints (e.g., Trippas et al., 2017). This state of affairs is partly due to the difficulties associated with fitting such models when individual data are sparse. But fortunately, some of these difficulties have been relaxed due to the development of hierarchical Bayesian extensions (e.g., Vandekerckhove et al., 2011).

### Stop worrying about data sparseness and embrace partial pooling

As discussed earlier, one of the challenges experimental psychologists regularly face is the sparseness of data. One obvious way to ameliorate this sparseness is to maximize the number of responses per individual. However, the notion that more data is necessarily better is a dangerous one, especially when dealing with higher-cognitive faculties. For instance, there is the risk that the way individuals engage syllogisms depends on their expected workload (e.g., number of syllogisms to be evaluated) throughout the experiment. For example, the studies by Klauer et al., (2000) relied on a large number of participants evaluating a small number of syllogisms each (as small as eight syllogisms). In contrast, studies with the goal of obtaining ROC data, such as virtually all of the studies we considered, involved larger numbers ranging from 16 to 64 syllogisms. It is possible that this difference can explain to some degree the discrepancies found in these studies regarding the effect of conclusion believability on participants’ discriminability. When relying on a hierarchical Bayesian approach, one can avoid a maximization strategy by capitalizing on the principle of partial pooling—that the similarities among participants will inform the estimation of individual-level parameters. The sparseness found at the individual level can be compensated for by a reliance on large participant samples that can be conveniently collected online, for example. The advantages of hierarchical Bayesian modeling would also hold in the case of *incomplete* experimental designs that attempt to sidestep time constraints, fatigue, learning, or carryover effects (Little and Rubin, 1997; Schafer, 1997). For example, partial pooling would improve parameter estimation in an experiment in which participants engage in different tasks and encounter different stimuli (e.g., Thompson, 2000), but not all participants engage in same set of tasks and/or encounter the same set of stimuli.

## Figures and Tables

**Fig. 11:**
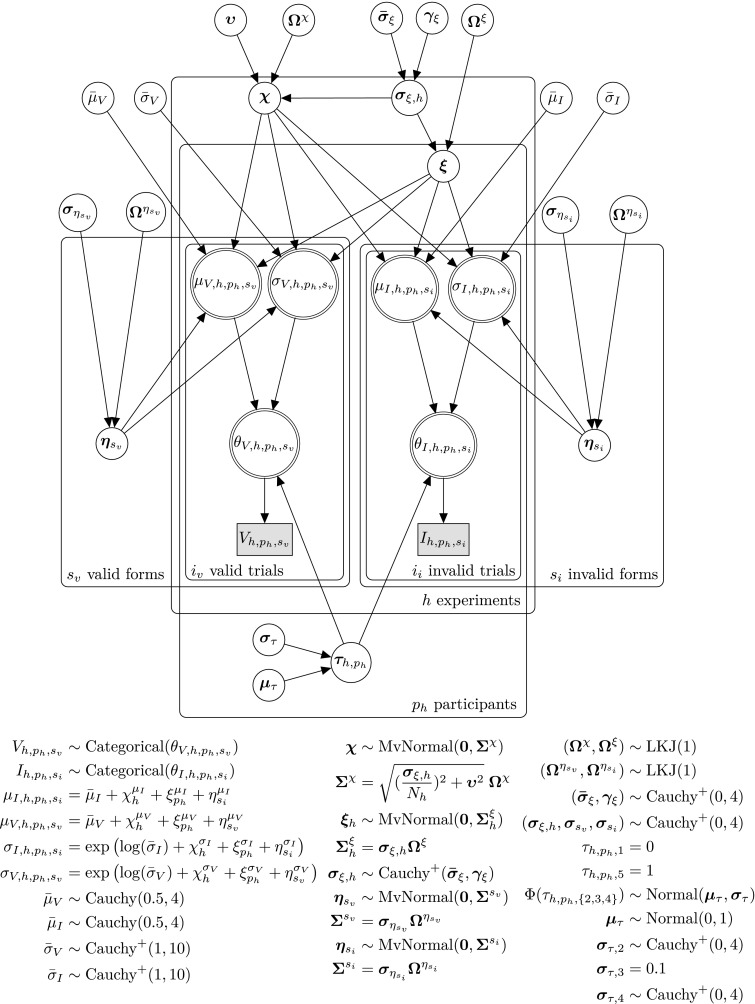
Graphical model of the hierarchical Bayesian SDT model

## Appendix A

### Details on the hierarchical Bayesian SDT model

A graphical representation of our hierarchical Bayesian SDT model is displayed in Fig. 11. This graphical representation follows the conventions used by Lee and Wagenmakers (2013): Discrete variables are displayed as squares and continuous variables as circles, the observed variables are displayed as shaded nodes, whereas the unobserved variables are non-shaded, and the double-bordered nodes represents variables that follow deterministically from other variables. Hence, all estimated variables are single-bordered, round, and non-shaded. Finally, the plates display the hierarchical structure of the model and all bold variables are non-scalar values such as vectors or matrices. Below the graphical model, Fig. 11 shows the distributional assumptions of our model as well as the complete prior structure. Note that in the figure the second parameter of Normal distributions is the standard deviation and not the variance. The only information missing is the exact specification of the SDT model which is given in Eqs. 16 and 17.

We used Hamiltonian Monte Carlo methods to explore the joint posterior distribution, as implemented in Stan (Carpenter et al., 2017). We used six independent chains with 1000 samples each, discarded the first 25% as warm-up samples (i.e., burn-in), and retained only every third iteration (i.e., thinning). Chain convergence was assessed visually for all hyper-parameters and by inspecting the Gelman-Rubin $\hat {R}$ statistics for all sampled parameters. The largest $\hat {R}$ for the main (i.e., “original”) model was 1.03 (i.e., below the 1.05 threshold). The largest $\hat {R}$ for any of the other model versions discussed in this paper was 1.05.

One distinctive feature of Stan is that it allows to separate a variance-covariance matrix $\boldsymbol {{\Sigma }}$ into a correlation matrix $\boldsymbol {{\Omega }}$ and standard-deviation vector $\boldsymbol {\sigma }$ with separate prior distributions. We employed this approach throughout and used completely non-informative priors for the correlation matrices, so-called LKJ priors with shape parameter 1 (Lewandowski et al., 2009; Stan Development Team, 2016). The priors for the variances were weakly informative, half Cauchy with location 0 and scale 4 (Gelman et al., 2013). Most of the remaining priors were also weakly informative Cauchy priors.

## Appendix B

### Probit regression analysis of study 14

We analyzed the data from Study 14 (Trippas et al., 2015) using a hierarchical Bayesian probit regression to assess whether the extent to which believability affects logical reasoning accuracy is moderated by individual differences in CRT performance (Frederick, 2005) when heterogeneity due to participants and stimuli is accounted for. The model can be specified in a straightforward fashion using the rstanarm package:

require(rstanarm)# next line implicitly requires the afexpackage afex::set_sum_contrasts() # sets-1/1 contrasts for factors globallyfit <- stan_glmer(rsp logic*belief*crt+(logic*belief|subj)+(belief|syll), family =binomial("probit"),data = study14)

where study14 is a data.frame in the long-format containing the variables subj, syll, rsp, logic, belief, and crt. A description of each variable is presented in Table 5.

**Table 5:** Description of the variables in the probit regression analysis

Variable	Description
subj	Identifier for each participant (factor)
syll	Identifier for each syllogistic structure (factor)
rsp	Response: 1 if endorsed as valid, 0 if rejected
logic	Logical Validity: valid or invalid (factor, first level = valid)
belief	Conclusion Believability: believable or unbelievable (factor, first level = believable)
crt	CRT-grouping: high or low (factor, first level = high)

The syntax can be interpreted as follows: the part before the tilde ˜specifies the outcome variable, in this case a binary accept/reject response rsp. The part after the tilde ˜specifies the fixed and random-effects. The fixed-effects specification logic⋆belief⋆crt corresponds to an intercept (grand mean), a main effect of validity, a main effect of believability, a main effect of CRT, a Logic $\times $ Belief interaction, a Logic $\times $ CRT interaction, a Belief $\times $ CRT interaction, and a Logic $\times $ Belief $\times $ CRT interaction. The random-effects are specified between brackets. Specifically, (logic⋆belief|subj) corresponds to a random per-participant deflection from the intercept, from the effect of Logic, from the random effect of Belief, and from the Logic $\times $ Belief interaction. A covariance matrix capturing these random effects is implied. Finally, (belief|syll) corresponds to a random per-syllogism deflection from the intercept and from the effect of Belief—once again together with a covariance matrix capturing these effects. Weakly informative priors were set for all effects, with a normal distribution with mean 0 and standard deviation 4 and 16 being assigned to the intercept and slope coefficients, respectively.

The analysis showed that there was a credible main effect of Logic, .42 [.33, .51], suggesting that people were sensitive to logical validity. There was also a main effect of Belief, .42 [.34, .49], indicating that arguments with believable conclusions were endorsed at a greater rate than arguments with unbelievable conclusions. There was no effect of the CRT on the overall endorsement rate, .04 [-.02, .10]. These main effects were qualified by several higher order interactions. There was a small but credible Logic $\times $ Belief interaction effect, -.05 [-.09, -.01] suggesting that people may have reasoned somewhat better for the arguments with unbelievable conclusions than for the arguments with believable ones. There was a comparatively large Logic $\times $ CRT interaction effect, .16 [.10, .21], indicating that participants classified as analytic reasoned better overall than their intuitive counterparts. The Belief $\times $ CRT interaction went in the opposite direction, -.10 [-.17, -.03], indicating that analytic reasoners were less likely to accept problems on the basis of conclusion believability than the intuitive reasoners. Finally, as discussed in the main text, the theoretically crucial Logic $\times $ Belief $\times $ CRT interaction was also credible, -.07 = [-.10, -.03], suggesting the Logic $\times $ Belief interaction differed between intuitive and analytical reasoners.

## Footnotes

1 Dube et al., (2010) explain their results in terms of a criterion-shift account (“it’s a response bias effect”). However, as shown in detail below and elsewhere (Wickens and Hirshman, 2000; Singmann & Kellen, 2013), this interpretation is not entirely justified due to an identifiability problem in their model. In the current article, we will therefore refrain from adopting this interpretation and only consider whether or not we find differences in discriminability between believable and unbelievable syllogisms.

2 It is worth noting that Klauer et al., (2000) also advocates the use of extended experimental designs that yield data that are to be fitted with an unconstrained model.

3 Recasting the EVSDT model as a probit regression model highlights an important identifiability issue in SDT. In $2\times 2$ designs involving two pairs of distributions, SDT cannot distinguish between a shift in responses bias from a shift of a pair of distributions. Specifically, note that $\beta _{B}$ can be understood as a shift in argument strength imposed on the distributions for believable valid and invalid syllogisms (for unbelievable syllogisms, $\mu _{I} = 0$ and $\mu _{V} = \beta _{L}$; for believable, $\mu _{I} = \beta _{B}$ and $\mu _{V} = \beta _{L} + \beta _{B}$), or alternatively, interpreted as a shift of the response criterion (for unbelievable syllogisms, $\tau = \beta _{0} $; for believable, $\tau = \beta _{0} + \beta _{B}$). For a detailed discussion on this issue, see Singmann (2014) and Wickens and Hirshman (2000). This identifiability constraint in SDT also implies that the interaction parameter $\beta _{LB}$ captures changes in discriminability, as discussed above for the linear model.

4 We generated SDT predictions for a six-point ROC by establishing five equally spaced $\tau $ criteria between -1.64 and 1.64 (these criteria lead to cumulative false-alarm rates ranging from .05 to .95 in equal steps). These predictions were then fitted with an SDT model using maximum-likelihood estimation (using the methods implemented in Singmann & Kellen, 2013).

5 Note that this linear model does not include the possibility of participant $\times $ stimulus interactions. Such interactions cannot be estimated in the present context because we only have one participant-stimulus pairing (Christensen, 2011). However, the absence of such correlations is not particularly troubling given that they are expected to have a reduced impact of parameter estimates (see Rouder et al., 2008).

6 Because we had no prior knowledge about the distribution of the study-specific variances, we assumed that the square roots of the variances (i.e., the standard deviations) follow a half-Cauchy distribution with location ${\bar \sigma _{\xi ^{x}}}$ and scale ${\gamma _{\xi ^{x}}}$ (we preferred the Cauchy over the normal distribution here because of the fatter tails of the former):

24 $$ \sigma_{\xi^{x},h} \sim \text{Cauchy}^{+}({\bar\sigma_{\xi^{x}}}, {\gamma_{\xi^{x}}}). $$

7 In the meta-analytic literature the between-study error variance is commonly referred to as $\tau ^{2}$. As we use $\tau $ to refer to a parameter of the signal-detection model we use $\upsilon ^{2}$ to refer to the between-study error variance.

8 For achieving model convergence (i.e., $\hat {R}$ values below 1.05 and no so-called “divergent transitions” that can appear in Hamiltonian Monte Carlo) we had to fix the standard deviation of the Gaussian distribution for the middle criterion to a small value (i.e., .1), as it otherwise tried to converge on 0 (which is an impossible value for a standard deviation). This suggests that there was little variability in the position of the central response criterion delineating “valid” and “invalid” decisions across participants.

9 Note that there are at least two different schemes for the four syllogistic figures. The scheme used here (and also by Klauer et al., 2000; Khemlani & Johnson-Laird, 2012) and another scheme used for example by Dube et al., (2010).

10 For reasons of numerical stability, we opted for testing a small range of values rather than a point estimate $\left (\frac {\sigma _{V}}{\sigma _{I}} = 1\right )$.

11 In Fig. 10, we did not include the syllogisms presented in Study 2. Because these syllogisms were only included in this study, it is difficult to completely disentangle their effects (captured by ${\eta ^{x}_{s}}$) from the observed study-specific difference (parameter $\chi ^{x}$) associated with Study 2. These parameter estimates are reported in our supplemental material is hosted on the Open Science Framework (OSF). Specifically at: https://osf.io/8dfyv/.

12 Due to the absence of participant-specific effects (and thereby estimates of the within-study variability) this model does not implement a ‘random-effects meta-analysis’. Instead, this is a simple hierarchical model with one multivariate-normal group-level distribution for the study effects.

13 One criticism is that the interaction index corresponds to a special case of a specific discrete-state model, namely a restricted two-high threshold model (Dube et al., 2010). However, it is important to keep in mind that the shortcomings of a specific discrete-state model do not necessarily generalize to the class of discrete-state models as whole. In fact, when a more appropriate two-high-threshold model that can account for ROC curvature is used Klauer and Kellen (2010), one obtains a characterization of the data that is similar to the SDT model’s (Klauer & Kellen, 2011).
